# Molluscs generate preferred crystallographic orientation of biominerals by organic templates, the texture and microstructure of Caudofoveata (Aplacophora) shells

**DOI:** 10.1038/s41598-024-63042-7

**Published:** 2024-06-12

**Authors:** X. Yin, J. D. Castro-Claros, E. Griesshaber, C. Salas, A. Sancho Vaquer, A. G. Checa, W. W. Schmahl

**Affiliations:** 1 Bruker, Beijing, Scientific Technology, Minhang District, Shanghai, 200233 China; 2 Department of Geo- and Environmental Sciences, Ludwig Maximillians University Munich, Munich, Germany; 3 Departamento de Estratigrafía y Paleontología, Universidad de Granada, 18071 Granada, Spain; 4 Departmento de Biología Animal, Facultad de Ciencias, Universidad de Málaga, 29071 Málaga, Spain; 5 Instituto Andaluz de Ciencias de la Tierra, CSIC-Universidad de Granada, 18100 Armilla, Spain

**Keywords:** Electron microscopy, Behavioural ecology

## Abstract

Caudofoveata are molluscs that protect their vermiform body with a scleritome, a mosaic of unconnected blade/lanceolate-shaped aragonite sclerites. For the species *Falcidens gutturosus* and *Scutopus ventrolineatus* we studied the crystallographic constitution and crystal orientation texture of the sclerites and the scleritome with electron-backscatter-diffraction (EBSD), laser-confocal-microscopy (LCM) and field-emission electron microscopy (FE-SEM) imaging. Each sclerite is an aragonite single crystal that is completely enveloped by an organic sheath. Adjacent sclerites overlap laterally and vertically are, however, not connected to each other. Sclerites are thickened in their central portion, relative to their periphery. Thickening increases also from sclerite tip towards its base. Accordingly, cross-sections through a sclerite are straight at its tip, curved and bent towards the sclerite base. Irrespective of curved sclerite morphologies, the aragonite lattice within the sclerite is coherent. Sclerite aragonite is not twinned. For each sclerite the crystallographic c-axis is parallel to the morphological long axis of the sclerite, the a-axis is perpendicular to its width and the b-axis is within the width of the sclerite. The single-crystalinity of the sclerites and their mode of organization in the scleritome is outstanding. Sclerite and aragonite arrangement in the scleritome is not given by a specific crystal growth mode, it is inherent to the secreting cells. We discuss that morphological characteristics of the sclerites and crystallographic preferred orientation (texture) of sclerite aragonite is not the result of competitive growth selection. It is generated by the templating effect of the organic substance of the secreting cells and associated extracellular biopolymers.

## Introduction

The Caudofoveata and the Solenogastres form the two classes of the mollusc clade Aplacophora. Up to now, the Aplacophora diversified to more than 400 mollusc species^1,2^. About 300 species form the class Solenogastres and about 140 species comprise the class Caudofoveata^2–11^.

Caudofoveata and Solenosgatres molluscs are marine organisms that live from sublittoral to abyssal environments^7–9,11^. These molluscs are small-sized (in most cases are 0.1 to 1 mm long) animals with a vermiform body shape and follow a benthic lifestyle. The Solenogastres possess a rudimentary foot and glide along substrate surfaces, while the Caudofoveata lack a foot and burrow within soft sediment^1,7,11–13^. Scheltema^14^ and Edlinger^15^ proposed that the vermiform body morphology results from adaptation of the Solenogastres to a gliding/climbing lifestyle and of the Caudofoveata to a burrowing life habit. Nonetheless, irrespective of the specific lifestyles, Aplacophora molluscs require high deformability and flexibility of their soft body as well as of their protective mineralized cover. These allow the Solenogastres to glide, climb and curl on rough surfaces and around skeletal elements and enable the Caudofoveata to move and burrow within muddy sediment^14,15^. It has been suggested^14,15^ that the development of a vermiform body morphology is connected to the reduction or/and complete loss of the foot, respectively.

The soft body of Aplacophora molluscs is covered by a mosaic of mineralized skeletal elements, the sclerites. These form a protective envelope around the soft tissue of the mollusc and comprise the scleritome. Caudofoveata and Solenogastres sclerites consist of aragonite and have elongated morphologies. The sclerites of Solenogastres molluscs are spicule-shaped and hollow^16^, those of the Caudofoveata are blade/lanceolate-shaped and are fully mineralized (this study and^1,2,5,7,11,17^).

Morphological, developmental, ecological and evolutionary aspects of Aplacophora molluscs have been studied in the past^1,3–5,7,10,11^. The mineralized envelope of the Aplacophora, the scleritome, remained, however, little investigated up to now, except for the studies of Haas, Ivanov and Scheltema, and Wendt et al.^17–20^. Based on light microscopy and SEM imaging Haas^18^ and Ivanov and Scheltema^19,20^ illustrate morphological aspects of Aplacophora sclerites. The authors show that sclerite shape, length and width vary for species of the different Aplacophora groups, families and genera. In a recent study, Wendt et al.^17^ use high-resolution SEM, TEM and AFM imaging techniques and illustrate surface topology of the sclerites and their sub-micrometer scale internal structure for the Caudofoveata species *Falcidens* sp. None of these studies consider the crystallographic aspects of Caudofoveata aragonite. This is the goal of our study. Electron backscatter diffraction (EBSD) is a technique that is well suited for the determination of mineral phase and crystallographic orientation of crystals. EBSD measurements enable the understanding of crystal arrangement patterns (the microstructure) in a material^21^ and disclose many structural properties of the investigated structural hard tissue^22–29^. With the latter information some aspects of the biomineralization process can be disclosed. With EBSD, we measured crystal assembly patterns for the scleritome of the Caudofoveata molluscs *Falcidens gutturosus* (Kowalevsky, 1901) and *Scutopus ventrolineatus* (Salvini-Plawen, 1968). We complement crystal orientation results with morphological results of the sclerites and of the scleritome; the latter were obtained with field emission scanning electron microscopy (FE-SEM), laser confocal microscopy (LCM) imaging, and high resolution computed tomography (HRCT).

We use the obtained crystallographic information in the sclerites of *Falcidens gutturosus* and *Scutopus ventrolineatus* sclerites to (1) demonstrate for *individual sclerites* the high co-orientation strength of the comprising aragonite crystallites, (2) prove the single-crystallinity of individual sclerites and (3) show the untwinned nature of sclerite aragonite. For the *scleritome* we show (4) the staggered packing of the sclerites in the scleritome and (5) illustrate the specific organization of sclerite aragonite in the scleritome. We demonstrate that (6) aragonite c-axis orientation is parallel to the elongation direction of the mollusc body. The latter is an interesting finding, as it contrasts to aragonite c-axis orientation of conchiferan mollusc shells. For these, aragonite c-axis orientation is, more or less, perpendicular to the outer surface of the conchiferan shell. At last, we discuss (5) texture and microstructure controls for the Caudofoveata scleritome and document that the latter are not generated by a physical crystal growth process, but solely by ultrastructural aspects of epithelial cells and associated biopolymers.

## Results

**Figure 1:**
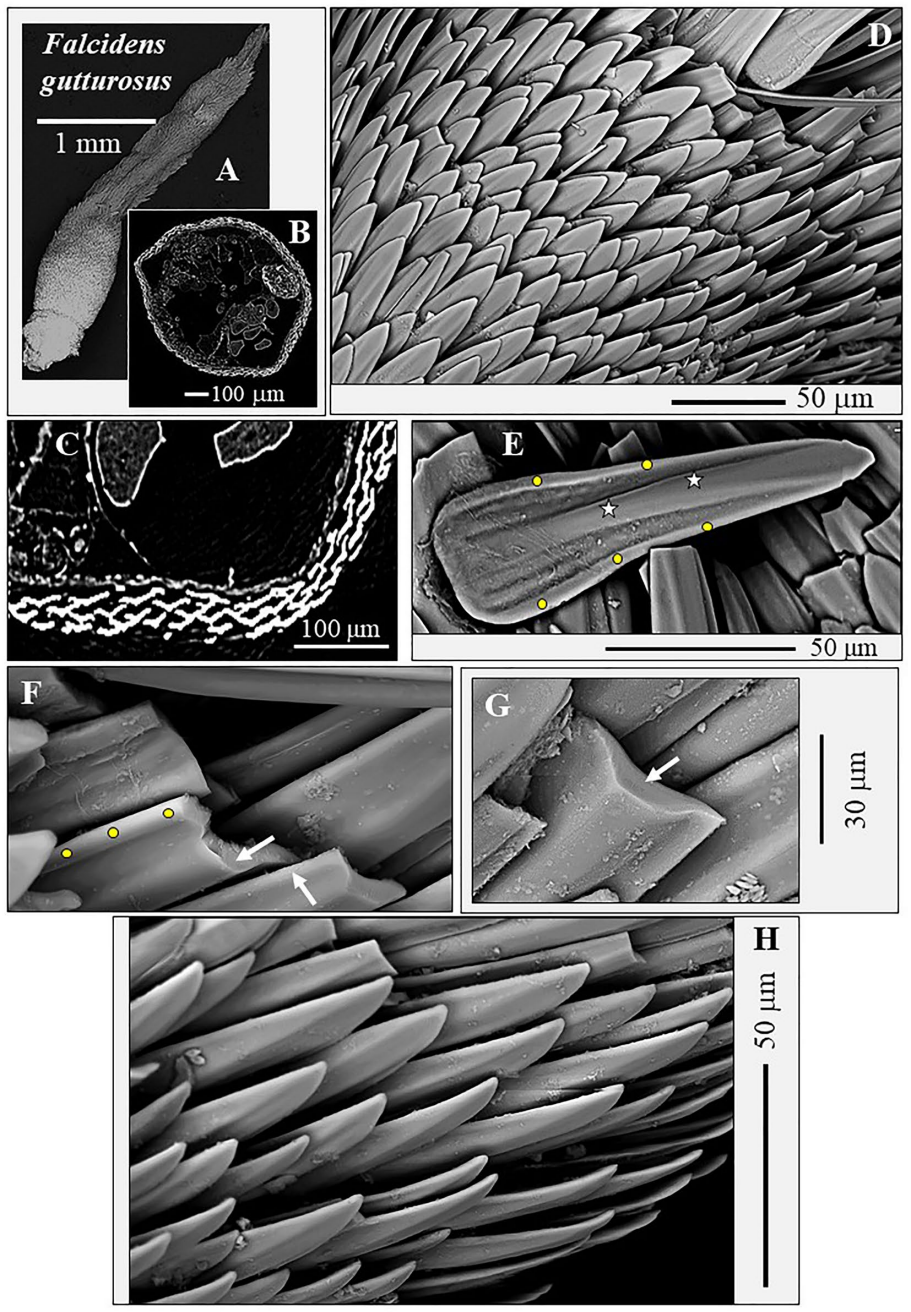
The scleritome that surrounds the cuticle of the Caudofoveata mollusc *F. gutturosus* (**A**). The envelope of sclerites consists of 5 to 6 rows of sclerites (**B**, **C**). Individual sclerites are blade-shaped (**D**, **E**, **H**); they are strongly thickened in their central part (white stars in **E**, white arrows in **F** and **G**) and their margins are bent upwards, away from the soft tissue of the organism (yellow dots in **E**, **F**). Accordingly, depending on the position of a cross-sectional cut through the sclerite, we observe curved to yoke-like sclerite cross-sectional morphologies (see also Figs. 2, 9). There is free space between adjacent sclerites, i.e. they are not solidly connected to each other (**D**, **H**). Nonetheless, their cross-sectional shape provides some degree of interlocking (white arrow in F and Fig. 2). (**A**) Laser confocal microscope image; (**B**, **C**) CT-scan micrograph. (**D** to **H**) SEM micrographs taken with BSE contrast.

**Figure 2:**
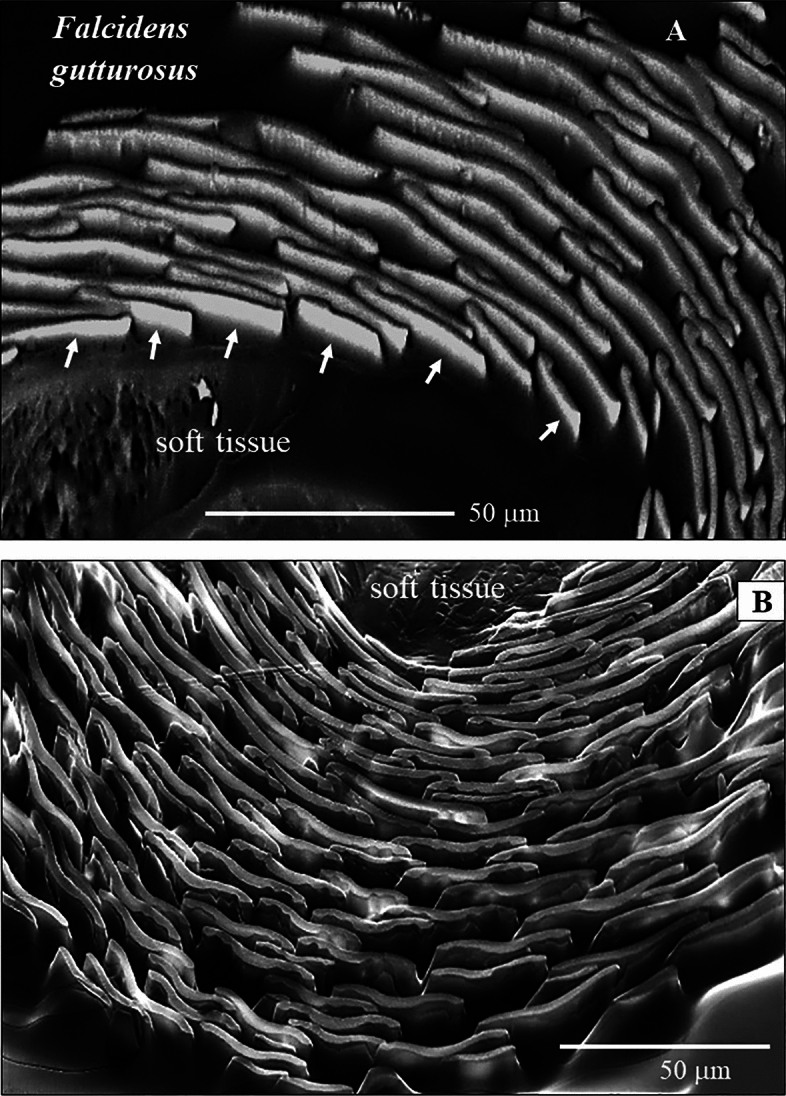
The arrangement of sclerites around the soft tissue of *Falcidens gutturosus*. (**A**, **B**) Transversely cut cross-section through the scleritome. (**A**) EBSD band contrast image; (**B**) SEM micrograph taken with BSE contrast. Next to the soft tissue of the organism, the sclerites show a staggered arrangement (**A**). Due to the thickened nature of sclerite center and rim, individual sclerites show, in cross-section, curved to bent morphologies (**B**) that provide some interlocking effect. The rim of sclerites curves away from the soft tissue of the organism (**B**).

**Figure 3:**
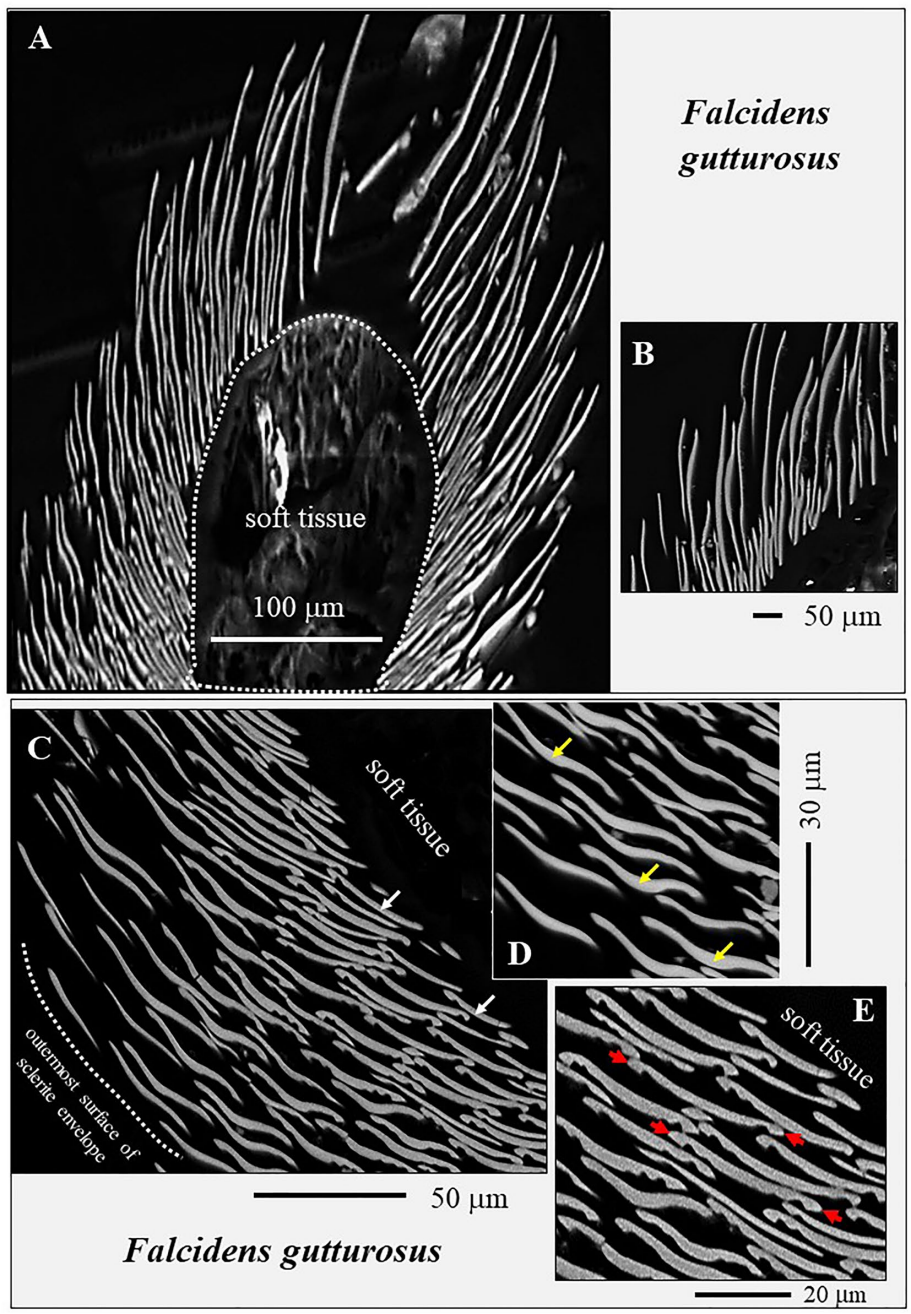
Longitudinal and cross-sectional morphology of sclerites that form the scleritome of *F. gutturosus* (**A** to **E**). (**A**, **B**) EBSD band contrast measurement images. (**C** to **E**) SEM images taken with BSE contrast. At the cuticle and soft tissue, we observe a dense row of small-sized sclerites, with sclerite length around 100 µm (**A**, **B**). These are suffused with longer-sized sclerites with lengths exceeding 200 µm (**A**, **B**). Cross-sections through the sclerites highlight their curved to yoke-shaped appearance in cross-section (white arrows in **C**), the thickening of their central portions (yellow arrows in **D**) and their upward curved margins (red arrows in **E**). The rim of the sclerites curves always away from the soft tissue of the organism (**C**, **E**). Sclerite rims have in cross-section a hook-like appearance (red arrows in **E**). This characteristic is very prominent for the sclerites of *F. gutturosus* and might facilitate the interlocking of the sclerites.

**Figure 4:**
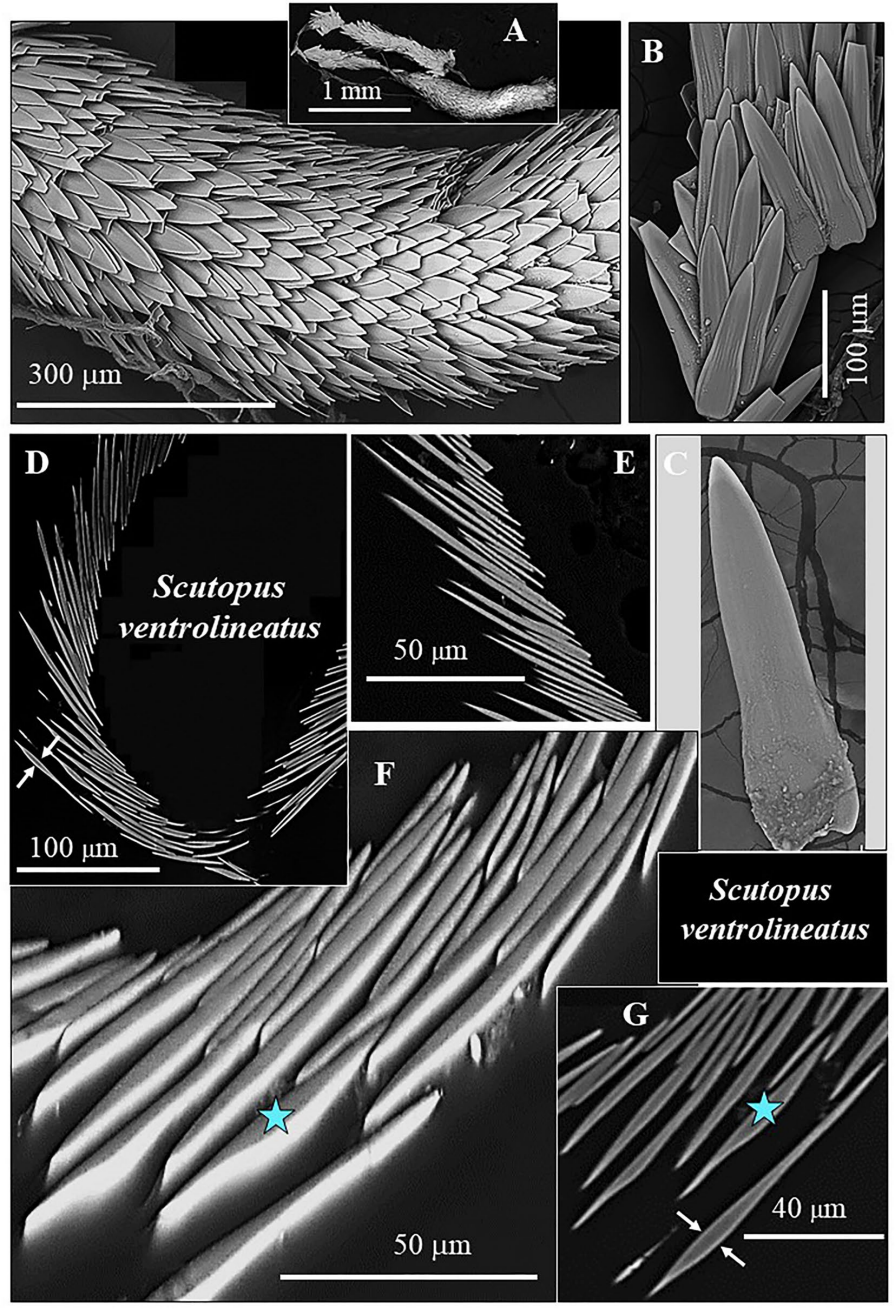
The scleritome that surrounds the soft tissue of the Caudofoveata mollusc *S. ventrolineatus* (**A** to **G**). (**A**, **B**, **C**, **F**) SEM micrographs taken with BSE contrast. (**D**, **E**, **G**) EBSD band contrast measurement images. The sclerites of *S. ventrolineatus* are, relative to the sclerites of *F. gutturosus*, thinner (**C**), less thickened in their central part (**C** to **F**), not as prominently curved along their margins and are rather lanceolate-shaped (**D**, **E** and blue star in **F** and **G**). Hence, in transversal cross-section, the sclerites have rather straight or only slightly curved morphologies (**C** to **G**). Adjacent sclerites do not touch (**A** to **G**) but overlap (**A**).

**Figure 5:**
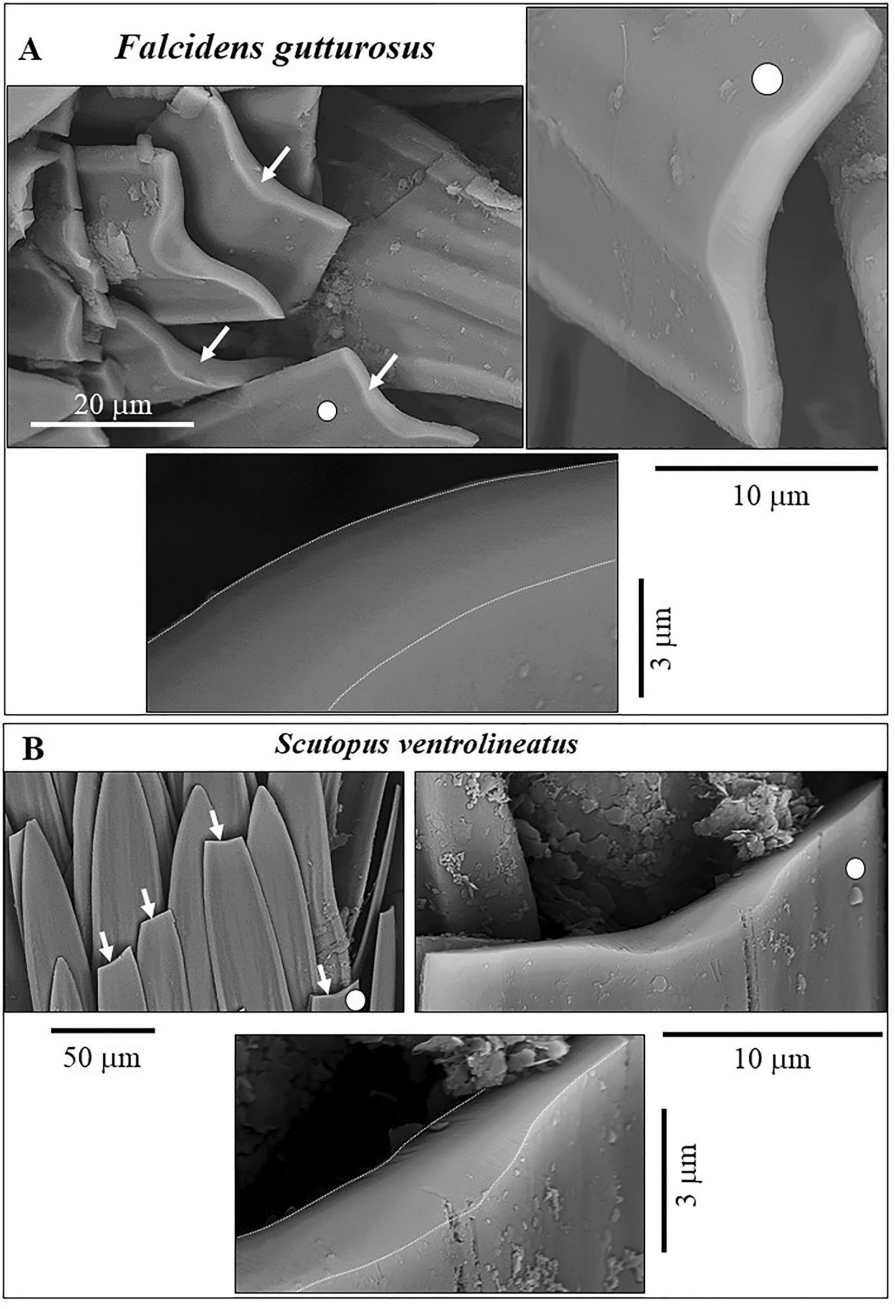
Fracture behavior of *F. gutturosus* (**A**) and *S. ventrolineatus* (**B**) sclerites. We see brittle fracture for the sclerites of both species and, accordingly, very smooth fracture surfaces. A conchoidal fracture with rough fracture surfaces is not detected, as observed for fractures through, e.g. brachiopod shell fibers. See Fig. S1 for an example of a conchoidal fracture and compare fracture surfaces shown in this figure and in Fig. S1.

**Figure 6:**
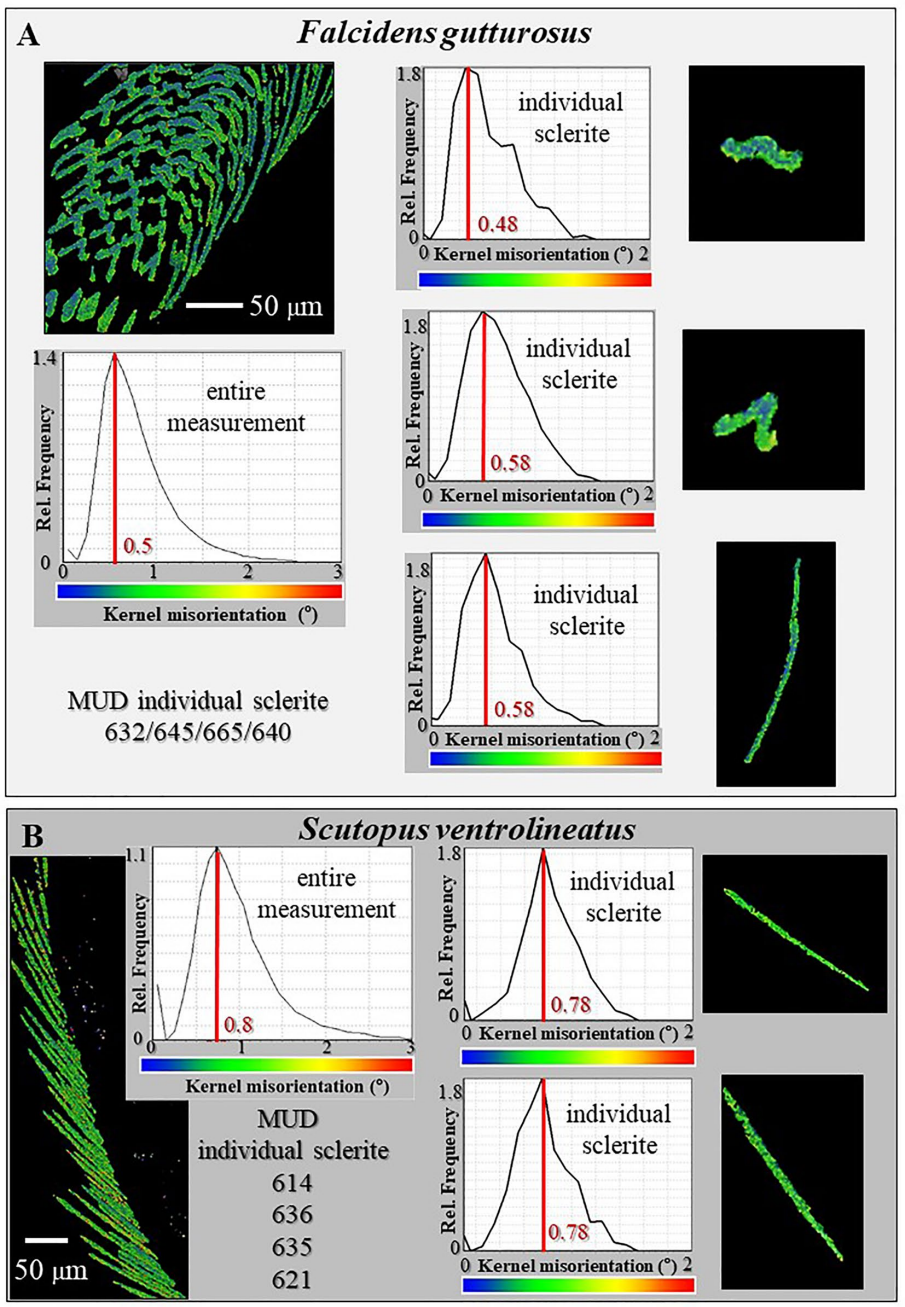
Crystal co-orientation strength for *F. gutturosus* (**A**) and *S. ventrolineatus* (**B**), individual and assemblies of sclerites. Kernel misorientation maps are given colour-coded and are complemented with corresponding relative frequency-misorientation diagrams. Kernel misorientation is calculated from EBSD data; the used colour-code for misorientation is given below the relative frequency—misorientation diagram. In addition, MUD values are listed for individual sclerites (**A**, **B**). Kernel misorientation as well as MUD values demonstrate that individual sclerites are almost single crystals. For an assembly of sclerites in the scleritome, Kernel misorientation is well below 3°, for individual sclerites Kernel misorientation is well below 2°. For both species, the maximal value for misorientation is below 1°. The latter scatters for *F. gutturosus* around 0.5°, for *S. ventrolineatus* around 0.8°. The aragonite in *S. ventrolineatus* sclerites is slightly more misoriented, relative to what is observed for *F. gutturosus* sclerites. This is also reflected by the MUD values for individual sclerites. For *F. gutturosus* MUD values of individual sclerites are slightly higher, relative to MUD values of individual sclerites for *S. ventrolineatus*.

**Figure 7:**
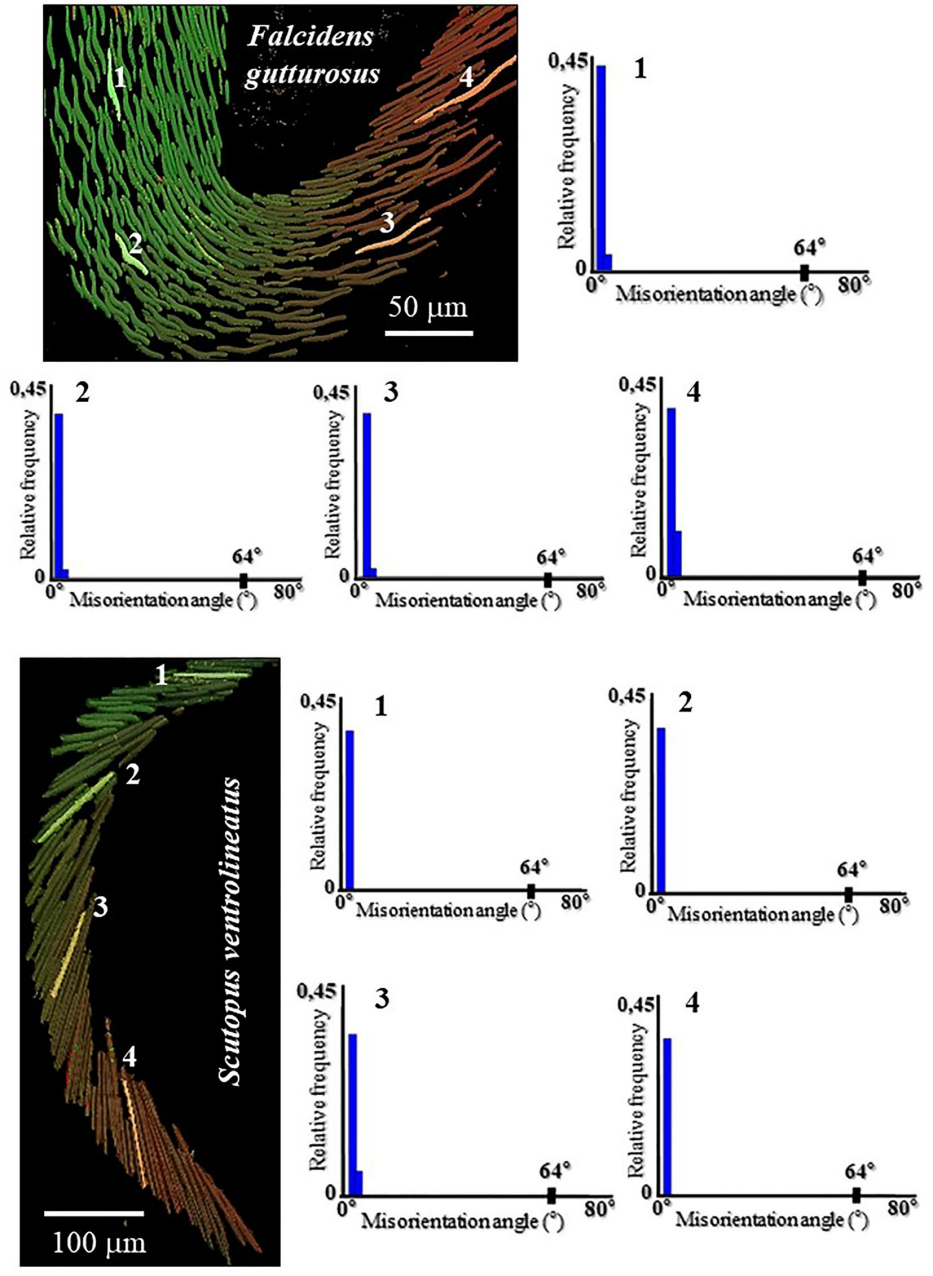
The absence of aragonite twinning in *F. gutturosus* and *S. ventrolineatus* sclerites. Relative frequency versus misorientation angle diagrams for individual sclerites, numbered 1 to 4. We show four diagrams per species. The chosen sclerites are highlighted in the relevant EBSD scan. For visualizing differences in orientation, the Euler colouring code is used, the coloring code is given in Fig. 12. We observe for the chosen sclerites a peak in misorientation angle below 5°, however, none at 64°. The latter (a peak at 64° misorientation) would indicate the presence of twinned aragonite in the sclerites. Due to the lack of the 64° peak, we can conclude that sclerite aragonite of *F. gutturosus* and of *S. ventrolineatus* is not twinned.

**Figure 8:**
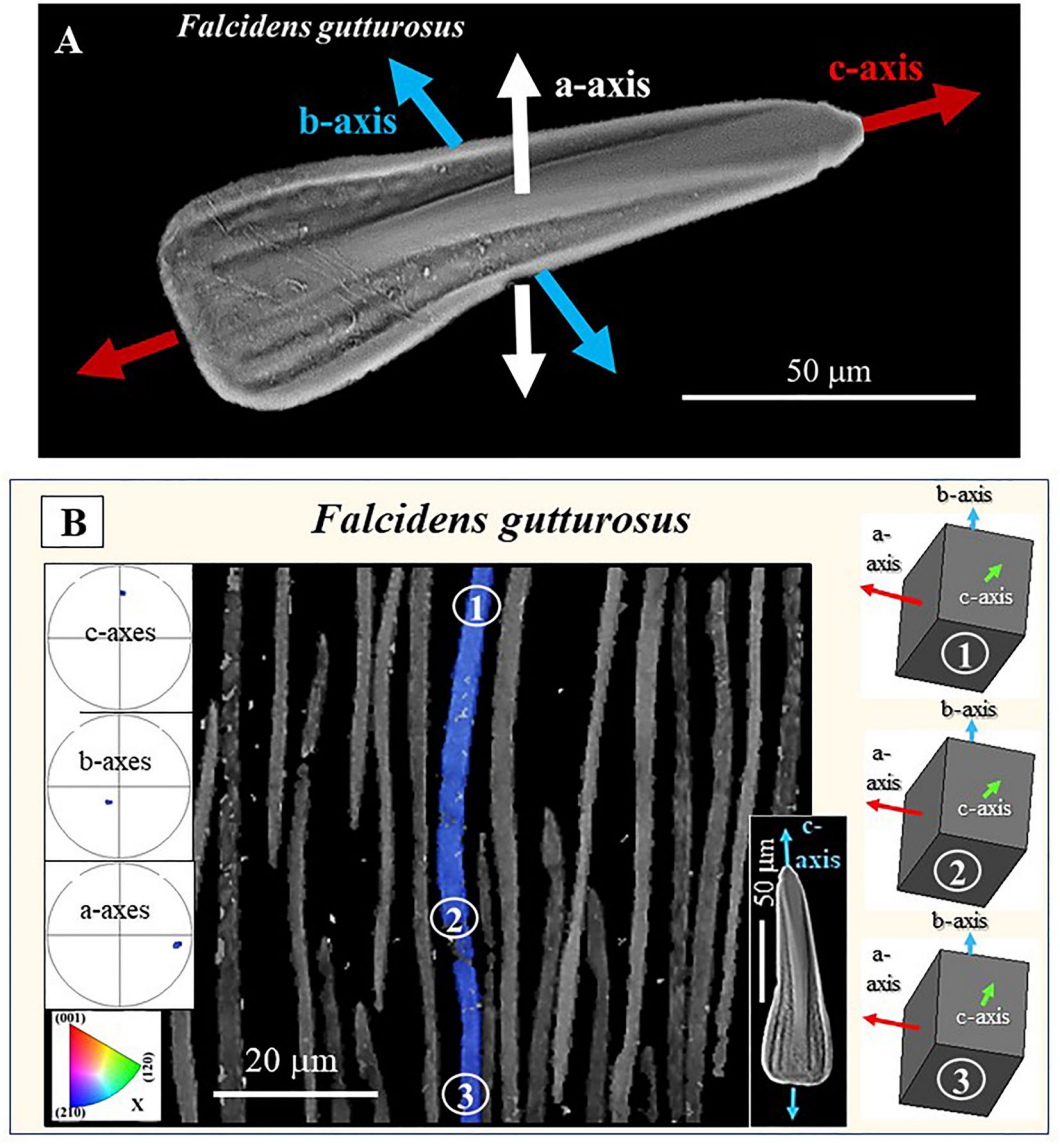
Orientation of aragonite crystallographic axes in individual sclerites. (**A**, **B**): *F. gutturosus*. (**A**) Aragonite c-axis is parallel to the long, the morphological axis, of the sclerite; aragonite b-axis is parallel to the width of the sclerite and aragonite a-axis is perpendicular to the main surface of the sclerite. A similar mode of aragonite axes orientation is observed for individual sclerites of *S. ventrolineatus.* (**B**) Aragonite crystallographic axes orientation in a longitudinally cut sclerite. EBSD band contrast measurement image (in grey), superimposed, for a selected sclerite, with the mode of crystal orientation (in colour). For that sclerite (given in colour in (**B**)), we show the mode of crystallographic axes orientation with sketched crystals and corresponding pole figures. The sclerite has a slightly curved appearance, nonetheless, aragonite lattice orientation is coherent (see the sketched crystals in (**B**)). Insert in (**B**): BSE image of a sclerite; blue arrows indicate aragonite c-axis orientation.

**Figure 9:**
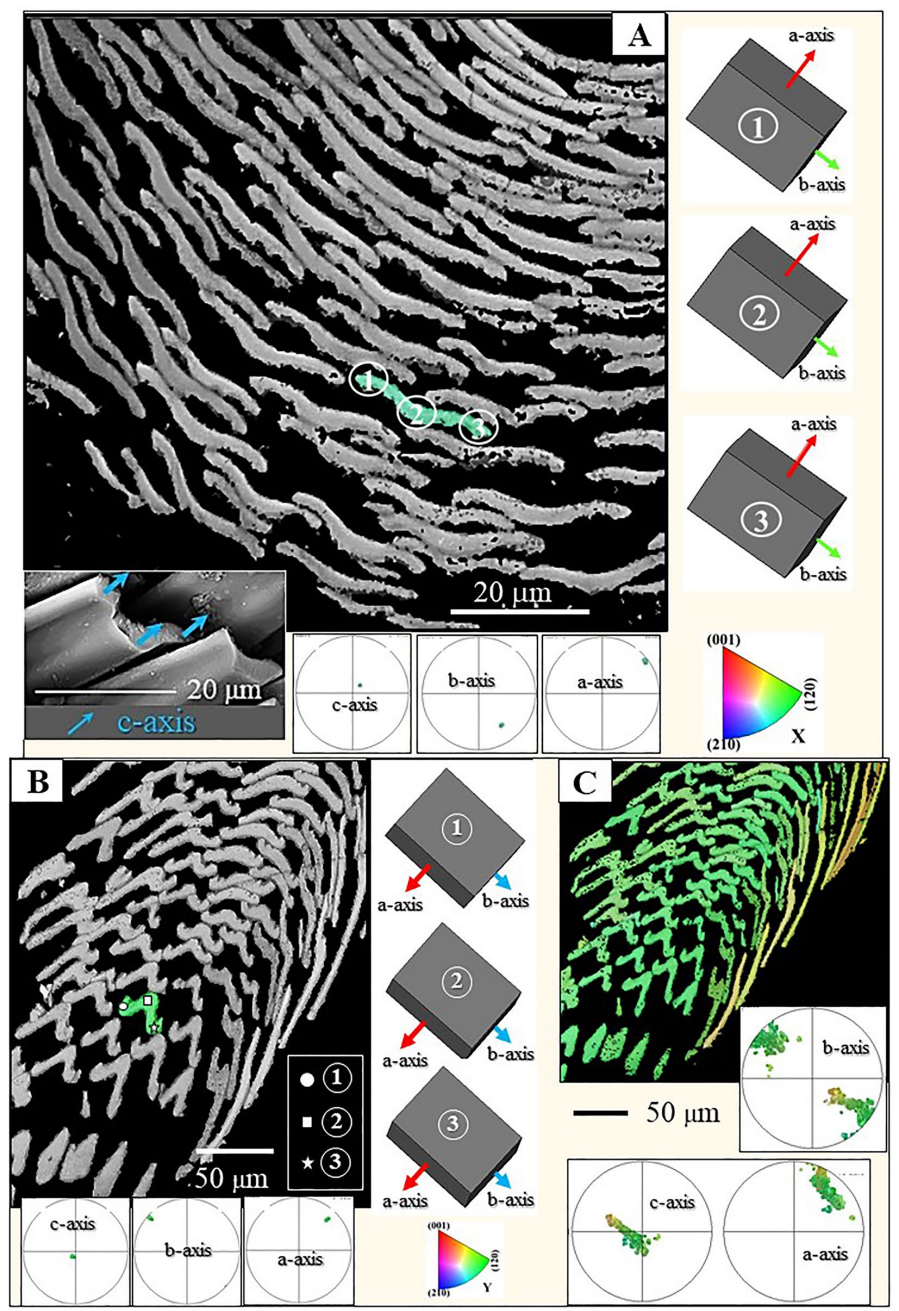
Aragonite crystallographic axes orientation of sclerites with curved morphologies. (**A**, **B**): *F. gutturosus*. (**A**): Transverse, (**B**) Diagonal cut through the sclerites and the scleritome. (**A**, **B**): EBSD band contrast measurement images (in grey), superimposed, for a selected sclerite, with the mode of crystal orientation (in colour). For this sclerite (in colour in (**A**, **B**)), we show the mode of crystallographic axes orientation with sketched crystals and corresponding pole figures. Even though, in cross-section, we find a curved sclerite appearance, aragonite crystallographic axes orientation does not change (see the sketched crystals). Hence, the aragonite lattice remains coherent. Insert in (**A**): BSE image of individual sclerites, blue arrows indicate c-axis orientation. The white dot, square and star and numbers 1, 2, 3 in (**B**) give the site where the orientation for the sketched crystals was taken. (**C**) Crystal orientation data for an entire EBSD scan taken on a large array of sclerites with curved morphologies.

**Figure 10:**
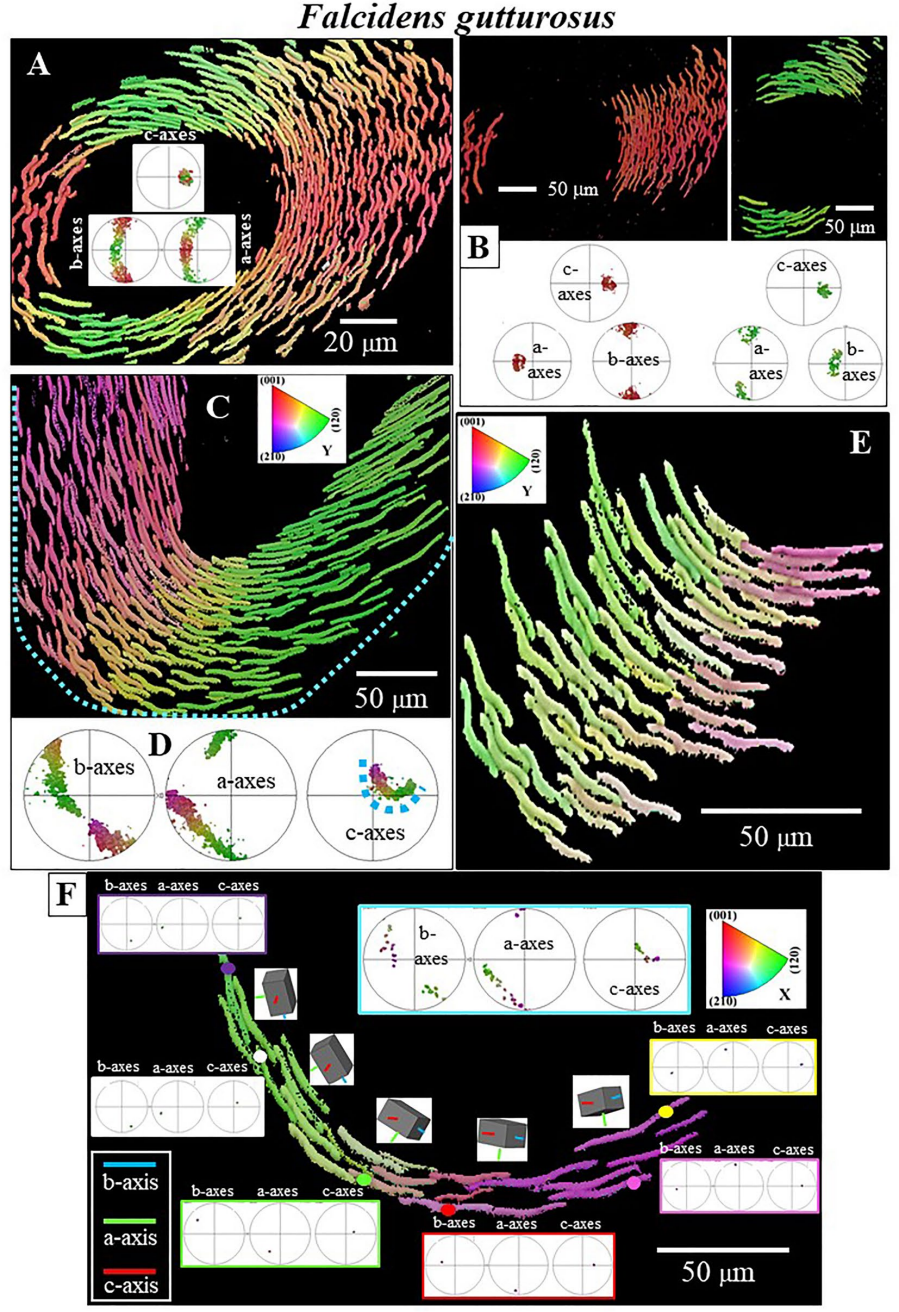
Mode of aragonite crystal orientation and pattern of sclerite organization in the scleritome of *F. gutturosus*. (**A**) to (**F**): Crystal orientation is shown with colour-coded EBSD maps and corresponding pole figures. Individual sclerites are single-crystals (Fig. 6) and are arranged around the cuticle and soft tissue of the organism in a strongly graded mode of organization. The single-crystalline nature of individual sclerites is obvious from the uniformity in colour of a particular sclerite, the graded pattern of sclerite arrangement is well visible from the smooth change of colour from one sclerite to the other (**A**, **C**, **E**, **F**). (**A**, **B**, **C**, **E**, **F**): Aragonite c-axis is perpendicular to the plain of view and parallel to the long axis of the sclerite.

**Figure 11:**
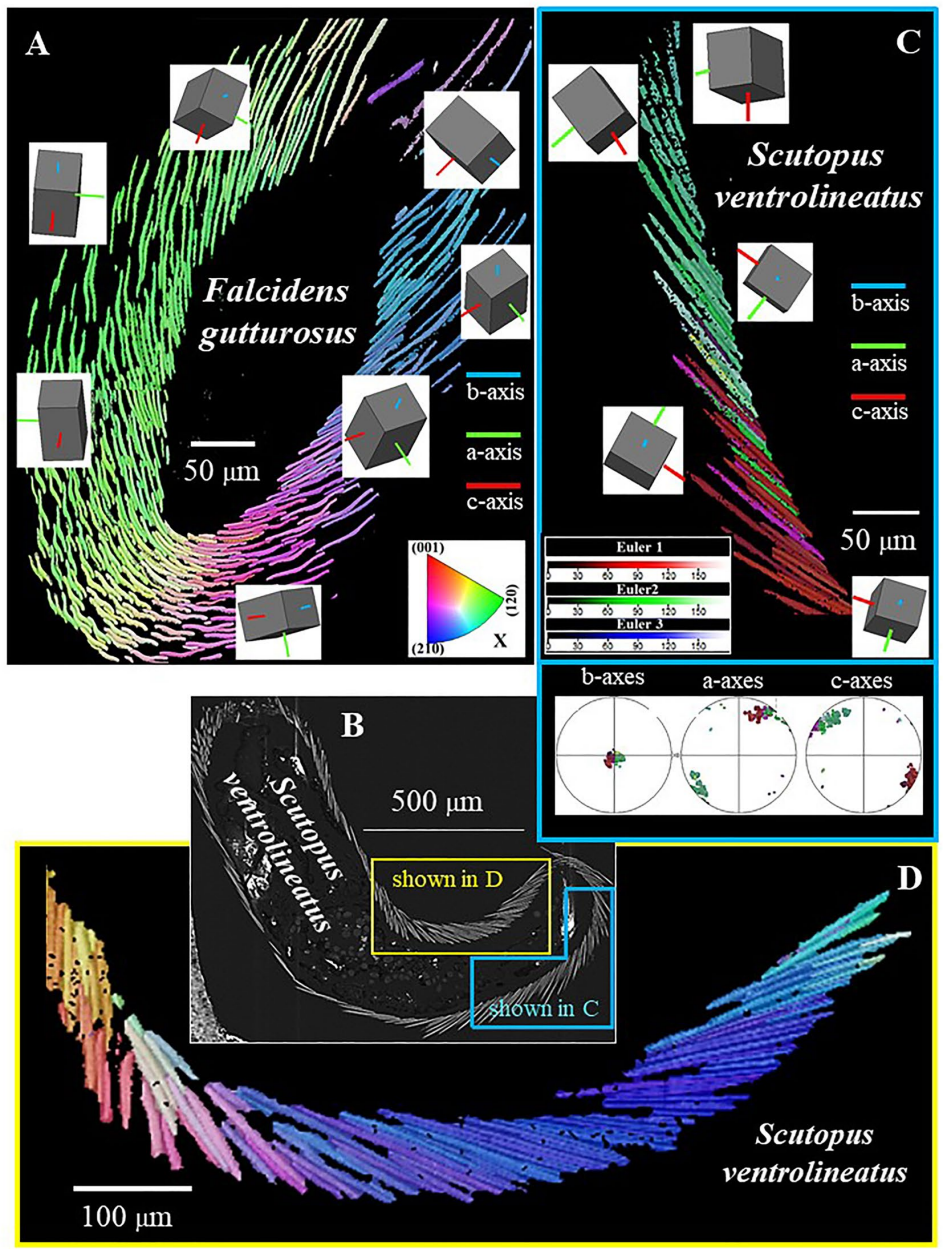
Aragonite crystal orientation and sclerite organization within the scleritome of *F. gutturosus* (**A**) and *S. ventrolineatus* (**B**–**D**). Crystal and sclerite arrangement is deduced from colour-coded EBSD scans. Sketched crystals in (**A**) and (**B**) point to the prevailing crystal orientation within the different portions of the scleritome. For both mollusc species it is well visible that individual sclerites are single crystals and that the sclerites are arranged in the scleritome with a gradual change of crystal orientation between neighboring sclerites.

**Figure 12:**
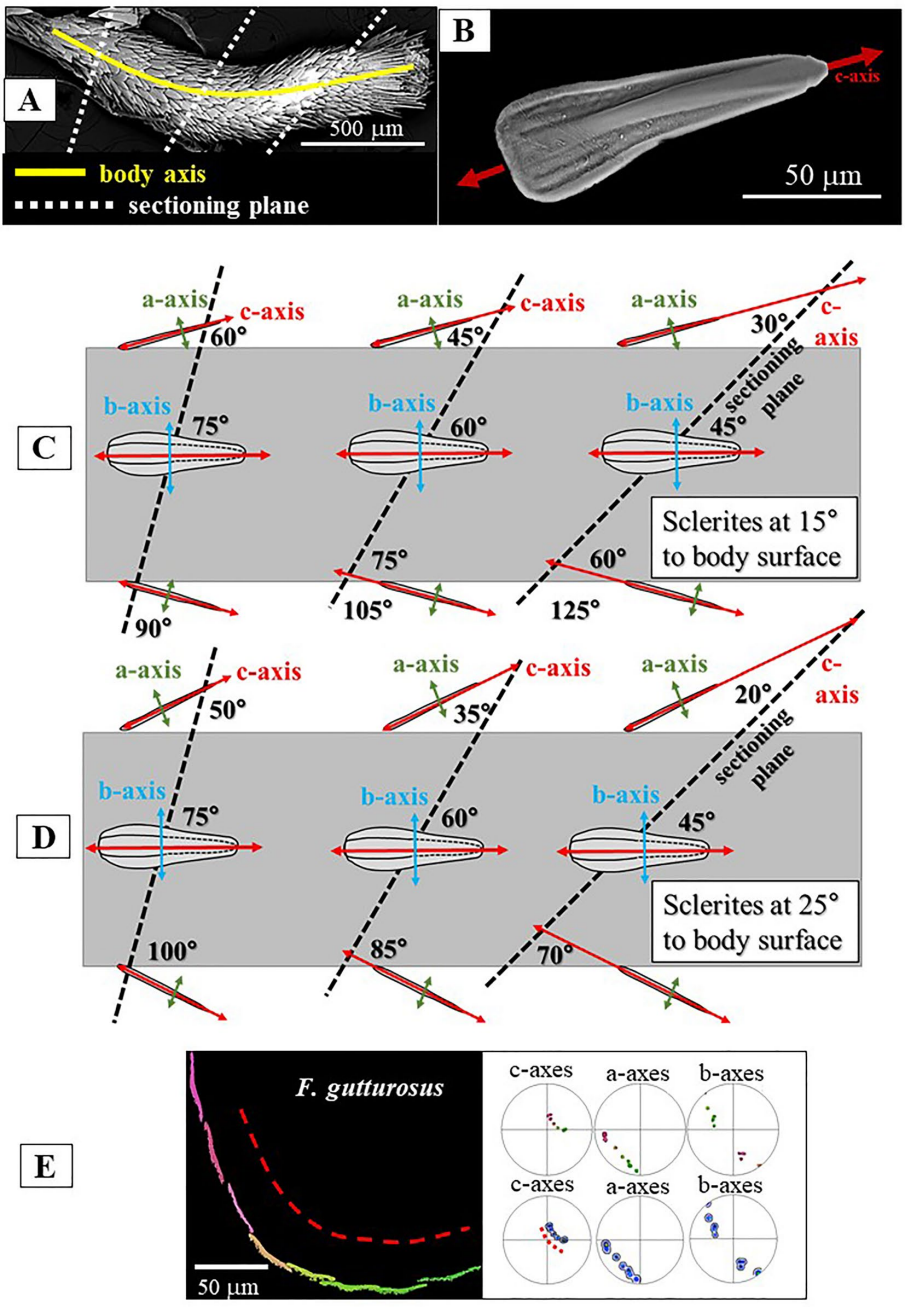
The effect of inclination variation of the scleritome when intersecting the sectioning plane. With an increase in inclination of the sectioning plane, the angles at which the c-axes of sclerites intercept with the top and bottom sides of the scleritome become successively different. The crystallographic a-, b- and c-axes of the sclerites are indicated with coloured arrows, according to the crystallographic model given in Fig. 8A. (**B**). Red: aragonite c-axis, blue: aragonite b-axis, green: aragonite a-axis. (**A**): SEM image of a *S. ventrolineatus* specimen with indication of the body axis and three sectioning planes with different orientations, relative to the scleritome: one perpendicular to the body axis and the two other sectioning planes with increasing inclination. (**C**, **D**): Simplified sketches of the mollusc body with the sclerites being inclined at 15° (**C**) and at 25° (**D**) to the mollusc body surface. (**E**) Subset of EBSD map shown in Fig. 10F. Only one row of sclerites is selected; note the smooth transformation of colour from sclerite to sclerite, demonstrating the graded mode of sclerite and aragonite c-axis arrangement. The effect of cut orientation influences the shape of c-axis distribution in the pole figure, see dashed red lines in the map and pole figure shown in (**E**).

We describe crystallographic and structural results of sclerite and scleritome aragonite for the Caudofoveata mollusc species *F. gutturosus* and *S. ventrolineatus*. Figures 1, 2, 3, 4 and 5 give general morphological features of the sclerites. Figures 6 and 7 demonstrate the single-crystallinity and the untwinned nature of individual sclerites (Fig. 6) and sclerite aragonite (Fig. 7). Figures 8 and 9 deal with aragonite crystallographic axes orientation in individual sclerites, and Figs. 10 and 11 describe the specific mode of aragonite and sclerite organization in the scleritomes of the investigated species. Figure 12 visualizes the variation of c-axis orientation for differently oriented cuts through the scleritome.

The investigated Caudofoveata species construct their scleritome of a highly structured arrangement of mineralized elements, a cover of blade/lanceolate-shaped sclerites (Figs. 1, 2, 3, 4). Next to the soft tissue of the mollusc, the sclerites are stacked in register (e.g. Fig. 2A). Adjacent sclerites overlap laterally and vertically (Figs. 1D, H, 4A) are, however, not attached to each other (Figs. 1H, 3A). The long axis of individual sclerites is at an angle to the long axis of the vermiform body of the mollusc. From Fig. 1D, H and Fig. 4A, D this angle can be estimated to be between 15° and 27°, with most frequent values being near 25°. Thus, for an exactly transverse cross section, the long axes of the sclerites form a cone with an opening angle of 15°–27° with respect to the long axis of the vermiform mollusc body. The observed opening angle and elliptic distortion of the cone in the pole figure depends on the orientation of the sectioning through the mollusc body (Fig. 12).

The sclerites of the investigated Caudofoveata species are thickened in their central part (Fig. 1E–G, arrows in Fig. 3D), relative to their marginal portions. The latter characteristic is most pronounced for the sclerites of *F. gutturosus*; for the sclerites of *S. ventrolineatus* we observe this feature as well, it is, however, less distinctive. Hence, depending on the position of a transverse cut through the blade/lanceolate-shaped sclerite, we observe in cross-section curved to corrugated sclerite morphologies (Figs. 1, 2, 3, 4). By comparison to the sclerites of *F. gutturosus*, the sclerites of *S. ventrolineatus* are thinner, less thickened in their central portion, more straight-lined, and show lanceolate morphologies (Fig. 4). The marginal portions of the sclerites of *F. gutturosus* are curved upward, always away from the soft tissue of the animal (Figs. 2B, 3C–E). In transverse section sclerite margins resemble a hook (arrows in Fig. 3E). This is not observed for the sclerites of *S. ventrolineatus*.

Even after being handled in the laboratory, only very few sclerites became fractured. The absolute majority of the sclerites in the scleritome remained fully intact (e.g. Fig. 4A). This indicates that individual sclerites are mechanically very resistent. However, for those sclerites that are fractured, the fracture is straight and not serrated (Fig. 5). We observe for the sclerites of both investigated species a brittle fracture behaviour with even fracture surfaces, and not, as it is the case for many biocarbonate hard tissues, e.g. for brachiopod shells, a conchoidal fracture behaviour and the development of conchoidal fracture surfaces (Figs. 5, S1).

Figure 6 shows Kernel misorientation maps and misorientation statistics for the scleritomes (left in Fig. 6A,B) and for individual sclerites (right in Fig. 6A,B). Kernel misorientation is obtained from EBSD measurements and gives the deviation in orientation between neighboring measurement points, thus, the misorientation between neighboring crystals or mosaic blocks. Neighboring crystals or mosaic blocks can have small-angle misorientations relative to each other, which are initiated by incorporation of substitutional ions or organic substance into the mineral. This leads to dislocations and small-angle grain boundaries^30–32^. There is very little difference in Kernel misorientation between the aragonite of an individual sclerite and an inorganic single crystalline analogue. For individual sclerites, maximal relative frequency values are between 0.48° and 0.78° in Kernel misorientation and misorientation scatters between 0.1° and 1.6°. For an inorganic single-crystal aragonite reference, maximal Kernel misorientation values are at about 0.4°/0.5°. Hence, we find very little difference in misorientation between sclerite aragonite and an inorganic aragonite reference.

Grain boundaries were not observed within individual sclerites. Accordingly, MUD values for individual sclerites are high and scatter between 600 and 670 (Fig. 6). MUD values of an inorganic single-crystal analogue are 700 or slightly above, when calculated with a half with of 5° and a cluster size of 3º (see the Methods section).

In essence, the small internal misorientations, the lack of grain boundaries and the high MUD values for individual sclerites reflect the single crystalline nature of the sclerites, an outstanding characteristic for biologically secreted carbonate hard tissues.

Figure 7 shows misorientation angle distributions for selected individual sclerites. These are highlighted in the EBSD maps shown in Fig. 7 and are numbered from 1 to 4. For individual sclerites we observe only small angle misorientations. The misorientation angle diagrams (Fig. 7) were calculated to determine whether sclerite aragonite is twinned or not. Twin formation is an important material characteristic, as it influences the physical properties of the material in question. A twinned crystal is a composite crystal of similar substance, consisting, however, of sub-crystals, the twin domains. The twin domains of a twinned crystal have different crystallographic orientations, however, the orientations are not random but are related to each other crystallographically, by a specific twin law. E. g. for aragonite, by a mirror plane, as it is the case for classical aragonite mirror twins on the {110} plane. The latter twin law for aragonite is equivalent to a ~ 64° rotation around [001]. Accordingly, if sclerite aragonite was twinned, a peak at 64° misorientation should be present in the relative frequency—misorientation angle diagram. As Fig. 7 shows, this is not the case, we do not observe a misorientation peak at 64° and we can conclude that sclerite aragonite is not twinned. This is an important finding, in particular, as biologically secreted conchiferan aragonites are very often twinned^33–38^. E. g. the aragonite of Polyplacophora spicules, scales and plates (belonging also to the clade Aculifera as the Aplacophora) is twinned^39^.

With EBSD measurements, we determine aragonite crystallographic axis orientation for individual sclerites (Fig. 8A). We find that aragonite c-axis is parallel to the morphological long axis of the sclerite. Aragonite a-axis is perpendicular to sclerite width and, correspondingly, aragonite b-axis, is within the width of the sclerite (Fig. 8A).

As shown in Figs. 2, 3 and 4, the sclerites of the investigated Caudofoveata species have a curved to bent cross-section (see also Figs. 8B, 9). This is, in particular, distinctive for the sclerites of *F. gutturosus*. Nonetheless, despite the curved shape of the sclerites, the crystallographic lattice orientation remains constant across the entire sclerite. We measure, across the entire cross-sections through a sclerite, similar aragonite a-, b- and c-axis orientation (see sketched crystals in Figs. 8B, 9). This is a result of the single crystallinity of individual sclerites. We observe the latter with the high MUD values for individual sclerites, low Kernel misorientation in the relative frequency—Kernel misorientation diagrams and unity in colour for crystallographic axes orientation for individual sclerites.

Figures 10 and 11 show aragonite crystal organization and sclerite arrangement in the *scleritome* of the investigated Caudofoveata species. For both species, irrespective of the slight differences in sclerite morphology, the most distinctive characteristic is the structured, gradual change of aragonite crystal orientation from sclerite to sclerite. The gradual change in aragonite orientation equals a rotation around aragonite crystallographic c-axis, the latter is sub-parallel (angles of 18°-27°) to the long axis of the mollusc body (Figs. 1A, D, 4A, 8A). The controlled change in aragonite orientation is well observable in sections perpendicular to the long axis of the vermiform mollusk body (Fig. 10) by the smooth transformation of colour between adjacent sclerites; in Figs. 10 and 11 the colour of the sclerites codes for crystal orientation.

Figure 12 illustrates cuts with different inclinations through the mollusc body. The orientation of the cut through the vermiform Caudofoveata body influences the shape of the approximately conical c-axis distribution in the pole figure, but, on a transverse cut through the scleritome (e.g. Fig. 10), it does not affect the smooth transition in crystal orientation from one sclerite to the other. As the long axes of the sclerites in the scleritome are sub-parallel (angle ca. 25°) to the length of the vermiform body of the mollusc, the ensemble of aragonite c-axes forms an approximately conical distribution around the long axis of the vermiform mollusc body. The opening angle and elliptic distortion of the cone in the pole figure depend on the inclination of the sectioning through the mollusc body (Fig. 12). The crystallographic orientation of the sclerites is directly coupled to their morphology (Fig. 12B). The orientation of the sclerites, both with respect to morphology and aragonite lattice orientation, is tied directly to the body surface and, thus, it follows the curvature of the latter. As a consequence, when following the circle of the body surface perpendicular to the body axis, the orientation changes gradually from one sclerite to the next. When following a line along the length of the body, sclerite orientation is constant as long as the body surface is not curved.

## Discussion

### The Caudofoveata scleritome

The Caudofoveata species that were investigated in this study surround their soft tissue with a 100 to 150 µm thick cover of aragonite sclerites. In cross-section, 4 to 5 rows of imbricated sclerites encase the cuticle of the molluscs.

Individual sclerites are free-standing elements and are only attached to the cuticle^40–44^. Sclerite arrangement around the cuticle is not random, it is structured. E. g. right next to the cuticle, the sclerites are arranged with a staggered organization pattern (Fig. 2A). Adjacent sclerites overlap but, do not interlock (Fig. 3). From a mechanical point of view this sclerite arrangement pattern is essential, as it secures considerable flexibility of the mineralized envelope at all movements of the animal. In essence, even though the mineral envelope is formed of rather small mineralized elements, their layered and overlapping assembly generates a firm, protective and highly flexible cover for the mollusc soft tissue.

Individual sclerites are sheathed by organic substance^44^. However, the aragonite is not within a continuous extracellular biopolymer matrix, as it is the case for the crystals of bivalve, gastropod and brachiopod shells^23^. A hard tissue, consisting of a continuous extracellular polymer matrix–mineral composite, would not be the appropriate cover for the Caudofoveata soft body. It would be too rigid for (1) their vermiform body shape and, especially, (2) for the mode of Caudofoveata movement, peristaltic locomotion, which is intrinsic for the Aplacophora^45^. As Caudofoveata molluscs are almost fully immersed within the substrate they need, on the one hand side, a protective cover that fully encases their soft body, that, on the other hand, renders a high degree of flexibility. This is accomplished with the assembly of detached but overlapping, flat, small, fully mineralized, stiff sclerites and their mode of organization in the scleritome.

Our results show two structural characteristics that are outstanding for the Caudofoveata scleritome: (1) the determinant of aragonite texture and (2) the controlled, graded, change of aragonite orientation from one sclerite to the other.

(1) Crystallographic preferred orientation of crystals in continuous mineralized tissue frequently gives the impression of being the result of growth selection of the constituting crystals^46,47^. An elaborate framework of theory and parametrization has been developed^48^ which compares the process of biological mineralization to solidification from melts, without taking into account the influence of the organic matrix on crystal orientation and texture formation. According to the concept of solidification from melts, the preferred orientation of crystals, their texture, is determined by the anisotropic growth speed of those crystals, where the fastest growth axis points towards the source of constituents (in the biological realm the mineralizing cells). For Ca-carbonate crystals, the axis of fastest growth is the c-axis. With progressive growth, this axis is the axis of the developing axial texture. The solidification from melts growth model presumes, at the start of mineralization, the formation of an initial layer consisting of randomly oriented small ‘seed’ crystals. With progressive growth, these crystals transform into large, columnar to prismatic, entities and the preferred crystallographic orientation of the fastest growth axis becomes parallel to the long axis of the columns and the prisms^46–48^. This growth model scenario qualitatively matches with the microstructure and texture of columnar/prismatic layers of eggshells^46,47^, brachiopods^49^ or molluscs^35,47–52^. It is, however, at odds with a number of other observations of biomineralized tissue. E.g., in the common fibrous microstructure of brachiopod shells, calcite c-axis is perpendicular to the fibres^53,54^, which is the slowest growth direction of calcite. In foraminifera^25–27^ and bivalve myostraca^35,38,55^ the initial fine-grained layer of seed crystals already has a strong axial texture. For the “single crystalline” sea urchin spines, an initial polycrystalline untextured layer has never been observed. In the case of the Caudofoveata, this initial polycrystalline, untextured, layer is also not evident in any of our measurements, and thus, most likely absent.

The most prominent examples, where crystal growth is not in accord with the solidification from melt model are the Caudofoveata (this study) and the Solenogastres scleritomes^16^. For the latter molluscs (1) adjacent sclerites in the scleritome are not attached to each other, are individual entities and (2) the sclerites, which are single-crystalline, do not have an axial texture, they have a single crystal texture; aragonite orientation within a sclerite is ordered in three dimensions. Accordingly, the growth selection, competitive growth, model for texture formation is not applicable for the Caudofoveata nor the Solenogastres scleritome (this study and^16^). The only feasible model for Caudofoveata and Solenogastres texture formation is epitaxial templating by the organic matrix, by the organic substance. The crystallographic preferred orientation pattern in Aplacophora scleritomes are so far the clearest examples for organic templating for texture formation as, for the Aplacophora, “inorganic” control by growth selection is out of question.

**Figure 13:**
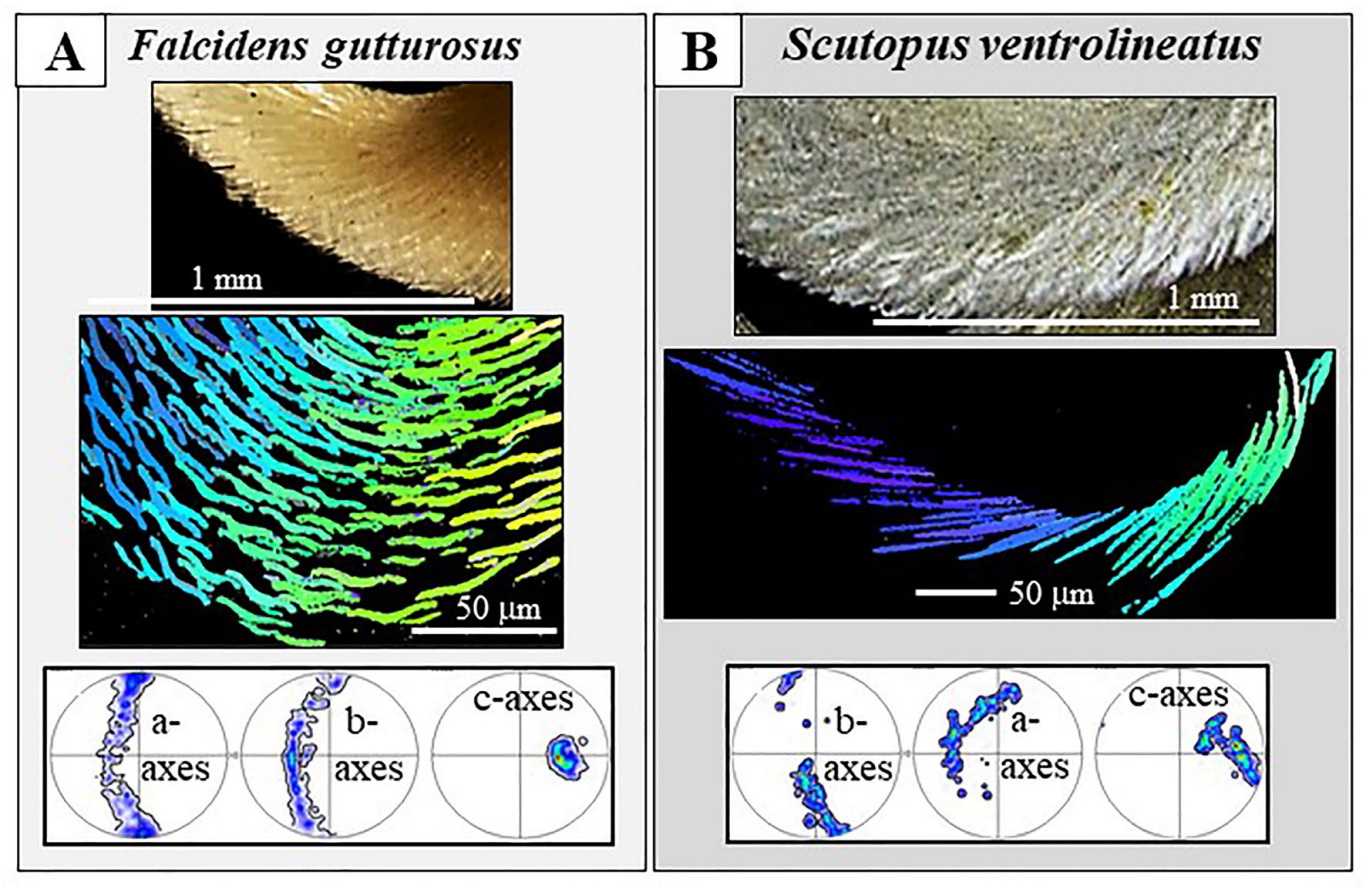
Summary of crystal arrangement characteristics for the scleritome of *F. gutturosus* (**A**) and *S. ventrolineatus* (**B**). Despite the difference in sclerite thickness and morphology, we observe for the sclerites of the two investigated mollusc species the following main structural characteristics: (1) the single-crystallinity of individual sclerites, (2) the strongly graded nature of sclerite organization in the scleritome and (3) the rotation of aragonite c-axis orientation with the curvature of the scleritome.

(2) The second outstanding microstructural characteristic of the Caudofoveata scleritome is the arrangement of the single crystalline sclerites, around the, in cross-section, circular Caudofoveata body (Figs. 10, 11, 13). For both, *F. gutturosus* and *S. ventrolineatus*, the sclerites assemble around the soft body with a controlled, a graded, tilt around sclerite aragonite c-axis (e.g. Figs. 10F, 12E). The crystallographic orientation of the sclerites is directly coupled to scleritome morphology (Figs. 1, 2, 3, 4). The orientation of the sclerites, both with respect to morphology and aragonite lattice orientation, is connected to the mollusk body surface and, as the Caudofoveata have a vermiform body morphology, follows the curvature of the latter. As a consequence, when following the circle of the body surface perpendicular to the long axis of the animal’s body, the aragonite/sclerite orientation changes gradually from one sclerite to the next. When following a line that is parallel to the mollusk body long axis, sclerite orientation remains constant as long as the body surface is not curved.

Controlled change of c-axis orientation with the curvature of the hard tissue is observed for many shelled organisms^56,57^. However, their crystallographic a-, and b-axes are usually randomly varying in orientation from crystal to crystal, resulting in an axial (cylindrical) texture. For Caudofoveata, the crystallographic orientation of sclerite aragonite is controlled in three dimensions, not only for a particular individual sclerite but also for neighboring sclerites. The crystallographic orientation of the sclerites is directly coupled to their morphology.

Such a stringently graded arrangement of adjacent crystals, as we find for the investigated Caudofoveata molluscs, has not yet been observed for any another biocarbonate hard tissue. The controlled gradation in crystal orientation is not simply the result of the vermiform body shape and its circular cross-section. The Solenogastres have similar vermiform body morphologies and the cross-section of their body is also circular. However, a gradation in aragonite crystallographic orientation is not observed for their scleritome^16^, as the arrangement of their sclerites in the scleritome is entirely different to that of the Caudofoveata.

Functionally graded materials with gradients in structure and composition are well-known from the biological as well as from the non-biological realm^58–64^. The advantage of functional gradients is that material properties can be varied locally, hence, only for a particular material volume, and not for the bulk structural tissue^63,64^. This ensures site-specific material property enhancements for site-specific functionality requirements, as it is, e.g. necessary for peristaltic locomotion, the type of locomotion used by the Aplacophora^45^. Soft-bodied burrowing animals employ peristaltic locomotion when they burrow or/and move through soft substrate^65–67^. This is also the case for the Caudofoveata^45,65^. Caudofoveata molluscs live almost completely buried within the substrate; only the caudal pair of the gills is sticking out of the substrate^11,15^. For peristaltic locomotion, the Caudofoveata use circular and longitudinal muscles, which allow them to form waves of elongation and shortening. These propagate along their vermiform body and induce that reaction forces become mobilized, which push the organism body forward^45,66,67^. In the case of the Caudofoveata, the anterior part of the body burrows into the substrate by active use of muscles^45,65^. The latter is supported by posterior sclerites which, at expansion of the animal, are pushed deeper into the burrow walls^65^. The latter mechanism anchors the Caudofoveata body within the burrow^65^. The arrangement of the sclerites serves as frictional asymmetry, similar to some asymmetrical ribs in bivalves, which are used by bivalves for the same purpose, namely for burrowing into soft substrate. The type of Caudofoveata movement and fixation of the body in the burrow requires local variation of material properties of the scleritome. This is best provided by the graded arrangement pattern of strongly mineralized and stiff sclerites. Such a strict gradation in crystal arrangement has not yet been observed for another biologically secreted carbonate hard tissue and is very specific. The scleritome of the investigated Caudofoveata mollusc species can be addressed as a functional graded biomaterial.

In essence, even though being footless^1^, Caudofoveata molluscs move within soft sediment. For the latter lifestyle the worm-shaped molluscs developed the right protection of their soft body. Their scleritome consists of a few rows of tessellated, thin, platy, fully mineralized, stiff, sclerites that are detached from each other, and show a graded arrangement of aragonite crystallites and aragonite single crystalline sclerites around the soft body of the mollusc. This is the right mineral cover for: (1) movement, (2) protection, (3) formation of local bending stiffness, and (4) maintenance of constant c-axis orientation, relative to the body surface of the mollusc. Accordingly, the Caudofoveata scleritome has an outstanding structural design, in particular when the restrictions, which are imposed by the mode of sclerite secretion, are taken into account^37^.

## The *Caudofoveata* and the *Solenogastres* sclerites and scleritomes

### The sclerites

Crystal orientation of individual sclerites and sclerite organization in the scleritome has been investigated for Solenogastres molluscs as well^16^. The comparison of morphological, structural and crystallographic characteristics of individual sclerites identifies for Caudofoveata and Solenogastres species many similarities and only few differences.

*Similar are*:

1. the carbonate mineral phase
2. the sclerites are individual elements, detached from each other,
3. the sclerites are only attached to epithelial tissue and papillae
4. the 3D appearance of individual sclerites, being thin and long
5. the single-crystallinity of individual sclerites
6. the direction of aragonite c-axis orientation parallel to the morphological, long, axis of the sclerite
7. the consistency of aragonite crystal lattice orientation for sclerites with bent, curved morphologies
8. the absence of twinned aragonite in the sclerites

*Different are*:

1. sclerite morphology (spicule-, or blade-shaped)
2. degree of mineralization (hollow, for many Solenogastres, or solid for the Caudofoveata)
3. mode of sclerite assembly (the microstructure and texture of the scleritome)
4. the direction of aragonite c-axis orientation, relative to the long axis of the mollusc body

A not yet resolved question concerns the phylogeny of the two Aplacophora mollusc classes; whether they are monophyletic, paraphyletic or separately derived. When based on anatomical studies, it is considered that the Solenogastres and the Caudofoveata are sister taxa, hence, constitute a monophyletic clade^13,14,68–70^. Taking, on the other hand, morphological aspects into consideration^1,7,70–75^, the results point to a paraphyletic nature of the Aplacophora, as the Aplacophora are considered to be two-class level taxa. However, molecular studies^2,76–82^ favour a monophyletic character for Aplacophora and do not support that the Solenogastres and the Caudofoveata are two independent basal groups.

Our crystallographic results do not allow us to conclusively support the one or the other phylogenetic assignment. Nevertheless, taking crystallographic aspects of sclerite aragonite into consideration, we observe for the sclerites of the investigated Caudofoveata (this study) and Solenogastres^16^ species significantly more structural similarities than differences. It should, however, be kept in mind that Solenogastres and Caudofoveata follow different lifestyles and live in distinct environments. And these impact structural features of individual sclerites and the sclerite organization in the scleritome as well.

Of particular interest is the single-crystalinity of Caudofoveata and Solenogastres sclerites, as, from a mechanical point of view, single-crystalline aragonite is not of advantage for a functional biological hard tissue. Biological tissues are functional materials that serve specific tasks for the sustainment of the organism. However, single-crystalline aragonite is a weak ceramic, it is brittle and breaks easily^83,84^. Hence, it is of little value as a construction material. For a structural biomaterial to be functional, the pure ceramic has to be functionalized, e.g. with addition of either impurities, e.g. polymers, or formation of specific microstructures^23,25,26,28^. Is this the case, then the result of biomaterial functionalization is a 20-30 times increase in strength and toughness, relative the single-crystalline analogue^83,84^. We find for Caudofoveata and Solenogastres sclerite aragonite lack of twinning, very high MUD values, very low Kernel misorientation and brittle fracture^16^. These characteristics do not indicate extensive incorporation of biopolymers into the aragonite, nor a specific toughness increase, due to an elaborate sclerite microstructure. We find that Caudofoveata sclerites are single crystals, a finding that is observed for the sclerites of Solenogastres molluscs as well^16^.

Hence, as single-crystalline aragonite is not of mechanical advantage, why are Aplacophora sclerites single-crystalline? Ultrastructural studies of Castro-Claros et al.^44^ demonstrate that individual Aplacophora sclerites are secreted by one cell only. Hence, individual mesodermal (for Caudofoveata) and epidermis (for Solenogastres) cells secrete, per cell, only one aragonite single-crystal, i.e. only one sclerite^44^. When crystals grow from solution, supersaturation is the driving force for nucleation and crystal growth^85,86^. At low supersaturation very few crystals form, while at high supersaturation nucleation is a catastrophic event and results in formation of very many crystals^85–87^. Crystals that grow at low supersaturation into a free volume develop well-defined crystal morphologies, while crystals that form at increased supersaturation have dendritic to spherulitic morphologies^88–90^. Geerken et al.^91^ and de Nooijer et al.^92^ have shown that foraminifera are able to vary cellular pH and, hence, supersaturation, at biocarbonate secretion. The single-crystalline nature of individual sclerites, their well-defined morphologies, lack of twin formation, and absence of other planar defects in the aragonite, might indicate that Caudofoveata and Solenogastres^16^ sclerites nucleate and grow at low supersaturation. Possibly, the restriction of sclerite aragonite to be single-crystalline is not for its mechanical properties. It is related to the necessity that only one sclerite should be secreted per cell, hence, it is a fabricational constraint. And this is an important requirement for the Aplacophora, as the molluscs need a, for protection mineralized, but, for their mode of movement flexible, scleritome. One way to realize protection together with flexibility is to generate a cover formed of individual, from each other, detached mineralized skeletal elements.

### The scleritomes

Aplacophora molluscs are benthic organisms^7–9,11^. The Solenogastres have a foot and glide along substrate surfaces, while the Caudofoveata lack a foot and are, however, well equipped to move within the substrate^1,7,11–13^.

**Figure 14:**
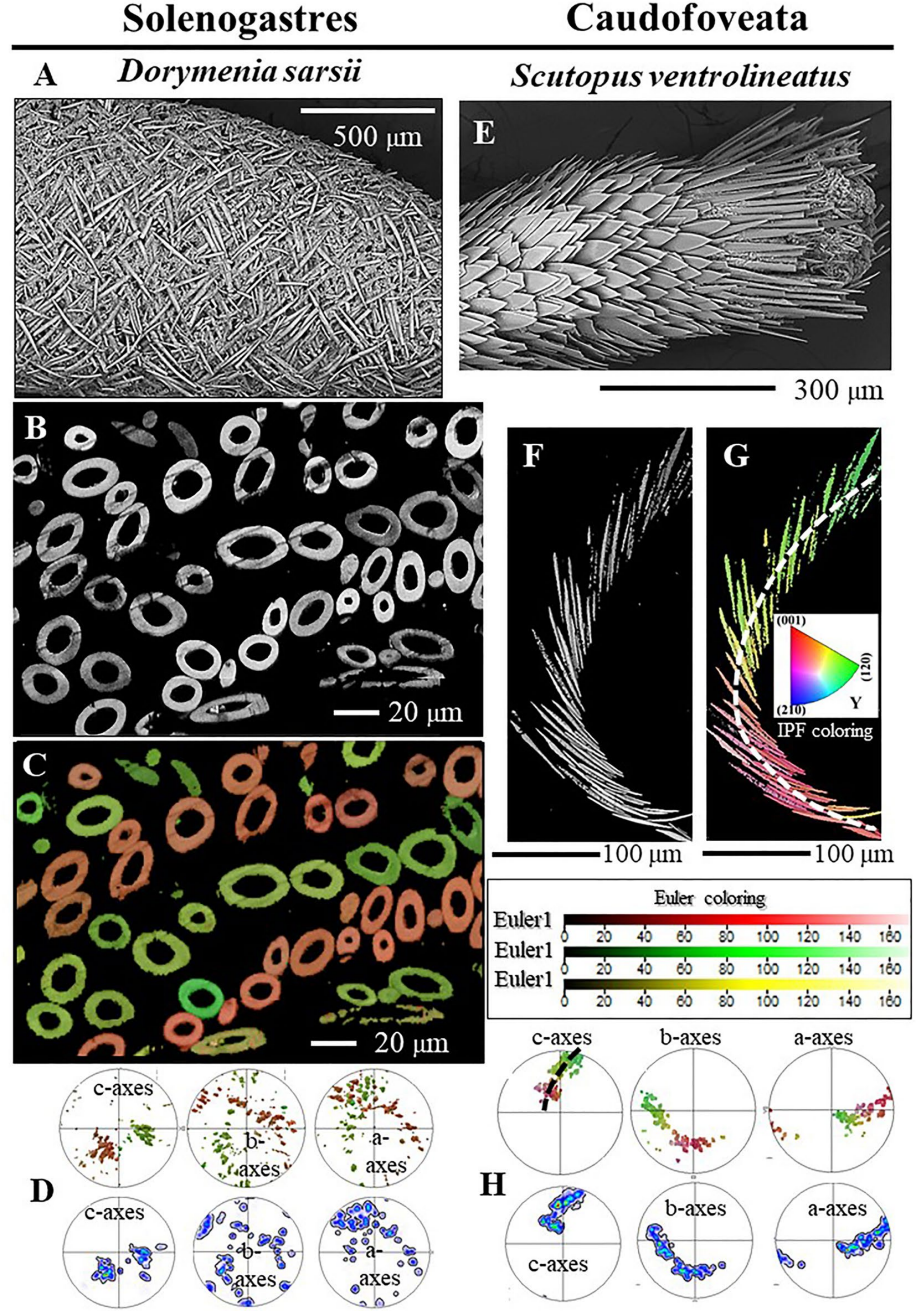
Juxtaposition of sclerite morphology (**A**, **E**), arrangement (**A**, **E**), aragonite microstructure (**B**, **C**, **F**, **G**) and texture (**D**, **H**) for Solenogastres (*Dorymenia sarsii*) and Caudofoveata (*Scutopus ventrolineatus*) molluscs. Crystal orientation shown in (**C**) is given with the Euler colouring mode, that in (**G**) with the IPF colouring code; for further information see the Methods section and^21^. Figure 13C is modified after Castro-Claros et al.^16^. We observe a significant difference for the species of the two Aplacophora classes in: (1) sclerite morphology, (2) arrangement in the scleritome as well as (3) mode of sclerite organization. Both mollusc species have vermiform body shapes and protect their cuticle with an envelope of sclerites. It is striking how different sclerite morphology and arrangement patterns are for the species of the two Aplacophora mollusc classes.

Figure 14 juxtaposes scleritome structure (Fig. 14A, E), microstructure (Fig. 14B, C, F, G) and texture (Fig. 14D, H) of a Solenogastres (*Dorymenia sarsii*) and a Caudofoveata (*Scutopus ventrolineatus*) mollusc species. For both molluscs, their soft body is covered by a meshwork of interlaced or imbricated mineralized elements. Hence, the overall structural principle of the *D. sarsii* (Solenogastres) and the *S. ventrolineatus* (Caudofoveata) scleritomes is similar. Nonetheless, as shown in Fig. 14, there is an immense difference between the Solenogastres and the Caudofoveata species in implementation of sclerite morphology, scleritome microstructure and texture. With the Caudofoveata and Solenogastres scleritomes, we find two different solutions of crystal, sclerite and scleritome organization for the realization of a mineralized cover enabling concomitantly rigidity and stiffness for protection, and flexibility and maneuverability for locomotion.

What causes the difference in Caudofoveata and Solenogastres sclerite morphology, scleritome microstructure and texture?

Difference in sclerite secretion?

Difference in lifestyle and environment?

Or both?

Castro-Claros et al.^44^ investigated for Caudofoveata and Solenogastres molluscs ultrastructural characteristics of the sclerite-secreting crystallization chambers. The authors^44^ show that the ultrastructure of the crystallization chambers is different for the species of the two Aplacophora mollusc classes. E. g., while microvilli seam the walls of Solenogastres crystallization chambers, they are absent at the crystallization sites of Caudofoveata sclerites^44^.

Furtheremore, Caudofoveata and Solenogastres molluscs inhabit different environments and follow different lifestyles. Even though body-shape and appearance in cross-section is quite similar, we find that morphology and internal structure of the sclerites as well as scleritome microstructure and texture are adapted to the different environments and lifestyles. *The scleritome of the gliding and climbing Solenogastres* consists of layers of interwoven, for most species, hollow spicules with densely mineralized spicule walls (Fig. 14A–C). Neither spicule arrangement nor crystal organization is staggered or graded, as it is the case for the Caudofoveata, shows, however, varying degrees of structuring (Fig. 14C, D and^16^). The *burrowing Caudofoveata body* is encased by layers of thin, platy, fully-mineralized, overlapping sclerites (Fig. 13E–G). The Caudofoveata scleritome is strongly structured and follows only one sclerite organization pattern, namely, the graded mode of sclerite assembly (Fig. 14F–H).

In conclusion, we can deduce for sclerite morphology and scleritome microstructure and texture an effect of both: the ultrastructure of the crystallization chamber as well as environment and lifestyle-related constraints.

## Concluding summary

Caudofoveata molluscs cover their vermiform body with an envelope of slightly corrugated, elongated, blade-shaped, fully-mineralized skeletal elements: the sclerites. The conjunction of the arrangement pattern of the sclerites in the scleritome and their morphological and structural characteristics render protection of the soft tissue from external threats at concomitant preservation of flexibility and movability.

In the current study, we focus on the crystallography of sclerite and scleritome aragonite and highlight and discuss structural characteristics of the scleritome of the Caudofoveata species *Falcidens gutturosus* and *Scutopus ventrolineatus*. The main findings and conclusions of our study are the following:

1. Up to 5 to 6 rows of imbricated sclerites envelope the cuticle of the investigated Caudofoveata mollusc species.
2. The sclerites are blade/lanceolate-shaped and are fully mineralized. Adjacent sclerites overlap laterally and vertically and surround the cuticle of the mollusc with a staggered arrangement pattern.
3. Individual sclerites are aragonite single-crystals.
4. Sclerite aragonite is not twinned.
5. The sclerites are at an angle of about 15° to 27° relative to the body of the mollusk and may act as barbs supporting peristaltic motion within the substrate. Accordingly, aragonite c-axis is at an angle of 15° to 27° (sub-parallel) to the long axis of the mollusc body.
6. For individual sclerites, aragonite c-axis is parallel to the morphological long axis of the sclerite. Aragonite b-axis is within the width of the sclerite, while aragonite a-axis is perpendicular to sclerite width.
7. The single-crystalline sclerites are arranged in the scleritome with a stringently graded mode of organization.
8. Internal structure, microstructure and crystallographic preferred orientation, the texture, of individual sclerites and of the scleritome are clearly not determined by inorganic physico-chemical controls (growth selection). They are generated by biological control, the templating effect of the organic substance of the secreting cells and associated extracellular biopolymers.

## Materials and methods

### Materials

We investigated the sclerites of the two Caudofoveata species: *Falcidens gutturosus* (Kowalevsky, 1901), and *Scutopus ventrolineatus* Salvini-Plawen, 1968 (Table 1). We investigated four specimens per species. One specimen per species was selected for high-resolution computed tomography (HR-CT). One specimen per species was selected for transmission electron microscopy (TEM) imaging and two specimens per species were selected for electron-backscatter diffraction (EBSD) measurements. Prior to preparation for EBSD measurements, one specimen per species was imaged with a laser confocal microscope (CLSM) and a scanning electron microscope (FE-SEM). EBSD measurements were performed on adult species and on various parts of the scleritome. In total, we performed 6 EBSD scans per species.

**Table 1:** The investigated Caudofoveata species together with sampling locations, the conducted expedition and date of sampling.

Species	Class	Family	Sampling location	Expedition sample date
Falcidens gutturosus	Caudofoveata	Chaetodermatidae	35° 54.07′ N–03° 01.61′ W 35° 53.81′ N–03° 01.41′ W	Expedition INDEMARES ALBORAN 22/09/2011
Scutopus ventrolineatus	Caudofoveata	Limifossoridae	36° 38.33′–3° 29.36′ W 36° 38.36′ N–3° 29.46′ W	Expedition ALSSOMAR 17/09/2019

The taxonomy of the studied species follows WoRMS Editorial Board (2024). World Register of Marine Species. Available from https://www.marinespecies.org at VLIZ. Accessed 2024-01-27. doi:10.14284/.

### Methods

#### Light microscopy and scanning electron microscopy imaging

Samples were imaged first with a Keyence 3D laser scanning microscope (VK-X1000 series) and an FE-SEM (Hitachi SU5000). Subsequently, samples were prepared for EBSD measurements.

### High resolution computed tomography (HRCT)

For HR-CT measurements, specimens were preserved in 100º ethanol and were post-fixed in OsO_4_ (2%) for 1 h at room temperature. Subsequently, specimens were dehydrated in a graded series of ethanol concentrations and, after that, were critical point dried. CT scans were performed with a Bruker SkyScan 2214-3D X-ray Microscope (voxel size = 0.36–1.1 µm) of the Central Services for Research Support (SCAI) of the University of Málaga, Spain, or with a Xradia 510 VERSA (ZEISS) microscope at the Centro de Instrumentación Científica (CIC) at the University of Granada, Spain. Images were post processed with the Dragonfly software.

### Electron backscattered diffraction (EBSD) measurements

For EBSD measurements, specimens were embedded in Sigma-Aldrich EPON resin or were immersed into Pattex superglue. Embedded/glued samples were trimmed, cut, and polished in a Reichert ultramicrotome with Diatome trimming, glass, and diamond knives. For EBSD measurements, the samples were coated with 4 to 6 nm of carbon. Measurements were carried out on a Hitachi SU5000 field emission SEM, equipped with an Oxford Instruments Nordlys II EBSD detector. At measurement, the SEM was operated at 15, 18 or/and 20 kV. Data were collected and evaluated using Oxford Instruments AZtec and CHANNEL 5 HKL software. EBSD measurements were performed with step increments of about 200 nm. For each species, we investigated two specimens. Each specimen was scanned with, 6 EBSD maps. Individual measurements lasted between 10 to 12 hours. EBSD scans were performed on different parts of the scleritome.

### Terminology

We use, in this contribution, the terms microstructure and texture in a crystallographic or/and material science sense. Crystallographic results were gained from electron backscatter (EBSD) measurements. EBSD is a highly suited analytical technique for the measurement of crystallographic axes orientations. For detailed information of the EBSD technique, see^21^.

The *microstructure* of a crystalline material is given by the arrangement pattern of crystallographic axes orientation of crystals. Hence, the microstructure of a structural material is the assemblage of 3D orientations of crystal lattices of the constituting crystals.

In this study, *microstructures* are presented with grey-scaled EBSD band contrast measurement maps as well as with colour-coded EBSD crystal orientation maps. The used colouring code is indicated in the figure or stated in the figure caption. For depicting differences in crystal orientation we use mainly the IPF colouring mode, in two figures (Figs. 7 and 13C) we use the Euler colouring mode^21^.

In crystal orientation maps, similar or distinct colours indicate similar or different crystal orientations.

Band contrast measurement images depict the backscattered signal strength in each measurement point. A high signal strength corresponds to light grey colours and indicates strong diffraction at the crystal lattice. Dark colours are indicative of non-diffracting substances, e.g. polymers, or an overlap of minute crystallites that cannot be resolved (indexed) automatically with the EBSD software^21^.

The *texture* of a crystalline material gives the nature of crystallographic axes orientation of crystals in a material. The texture of a material is the mode, the pattern of orientation of crystals^21^. In this study, the *texture* is presented with pole figures that give either the measured orientation data or the density distributions of these. For the density distributions, we use the lowest possible setting for half width and cluster size: a half width of five and a cluster size of three degrees. The half width controls the extent of the spread of the poles over the surface of the projection sphere, a cluster comprises data with the same orientation.

An *axial texture* is given when the c-axes show co-orientation (clustering in the pole figure around a single direction), while the corresponding a- and b-axes vary in orientation on a great circle perpendicular to the texture axis, in this case, the c-axis direction^21^.

Aragonite crystal *co-orientation strength* (given with MUD values) is derived from density distributions of the measured EBSD data^21^. The MUD (multiple of uniform (random) distribution) value is calculated with the Oxford Instruments CHANNEL 5 EBSD software. A high MUD indicates high crystal co-orientation strength, while low MUD values reflect low to negligible strength of crystallite or/and mineral unit co-orientation. With a half width of five and a cluster size of three degrees, an MUD of 1 indicates random orientation distribution and no preferred orientation, an MUD higher than 700 documents perfect crystallite co-orientation; a single-crystal-like co-orientation of crystallites^78,79^.

We process data gained from EBSD measurements for the visualization of *crystal misorientation patterns*: local Kernel misorientation. *Local Kernel misorientation* shows the deviation in orientation between neighboring measurement points, in this study, calculated for 3x3 clusters^21^. Misorientation results are given colour-coded, and the used colour-code is given with the Kernel misorientation figure. Deviation in orientation corresponds to local internal strain, e.g. caused by incorporation of biopolymers^30,93^.

We show relative frequency—misorientation angle diagrams. Data were calculated with the CHANNEL 5 software from EBSD scans. We observe a multitude of misorientations; these scatter between 5° and 100°. We do not observe a strong peak at 64°. The latter would be indicative of a systematic misorientation, e.g. a twin.

To index the aragonite EBSD patterns, we used the unit cell setting: a = 4.9614(3) Å, b = 7.9671(4) Å, c = 5.7404(4) Å^94^.

FE-SEM images taken with BSE contrast are obtained when incident electrons are backscattered into the vacuum from a comparatively deep location in the specimen. The backscattered electron signal covers the contrast which provides information about the compositional distribution of the specimen surface, e.g. if a polymer or a mineral is hit by the incident electron beam. In the latter case, one obtains differences in grey scale, e.g. the mineral is shown in bright grey, while the polymer is depicted in the image in dark grey. Furthermore, the backscattered electron signal contains also topographic information. However, this was not used in our study. We preferred SEM images taken with BSE signal, in contrast to micrographs taken with SE signal, as BSE images show very well the distribution of polymers and mineral in a sample.

### Supplementary Information

Supplementary Figure S1.

## Data Availability

The datasets generated during the current study are available from the corresponding author on request.
