# Supplementary material for: Molluscs generate preferred crystallographic orientation of biominerals by organic templates, the texture and microstructure of Caudofoveata (Aplacophora) shells
Source: Sci Rep. 2024 Jun 12;14:13469. doi: 10.1038/s41598-024-63042-7 (PMC11169368; doi:10.1038/s41598-024-63042-7)
Supplement: Supplementary file 1 — Supplementary Figure S1. [file 41598_2024_63042_MOESM1_ESM.docx]

**Molluscs generate preferred crystallographic orientation of biominerals by organic templates: texture and microstructure of *Falcidens gutturosus* and *Scutopus ventrolineatus* (Caudofoveata, Aplacophora)**

X. Yin^1, 2^, J. D. Castro-Claros^3^, E. Griesshaber^2^, C. Salas^4^, A. Sancho Vaquer^2^, A. G. Checa^3, 5^, W. W. Schmahl^2^

1. Bruker, Beijing, Scientific Technology, Minhang District, Shanghai 200233, China

2. Department of Geo- and Environmental Sciences, Ludwig Maximillians University Munich, Munich, Germany

3. Departamento de Estratigrafía y Paleontología, Universidad de Granada, 18071 Granada, Spain

4. Departmento de Biología Animal, Facultad de Ciencias, Universidad de Málaga, 29071 Málaga, Spain

5. Instituto Andaluz de Ciencias de la Tierra, CSIC-Universidad de Granada, 18100 Armilla, Spain


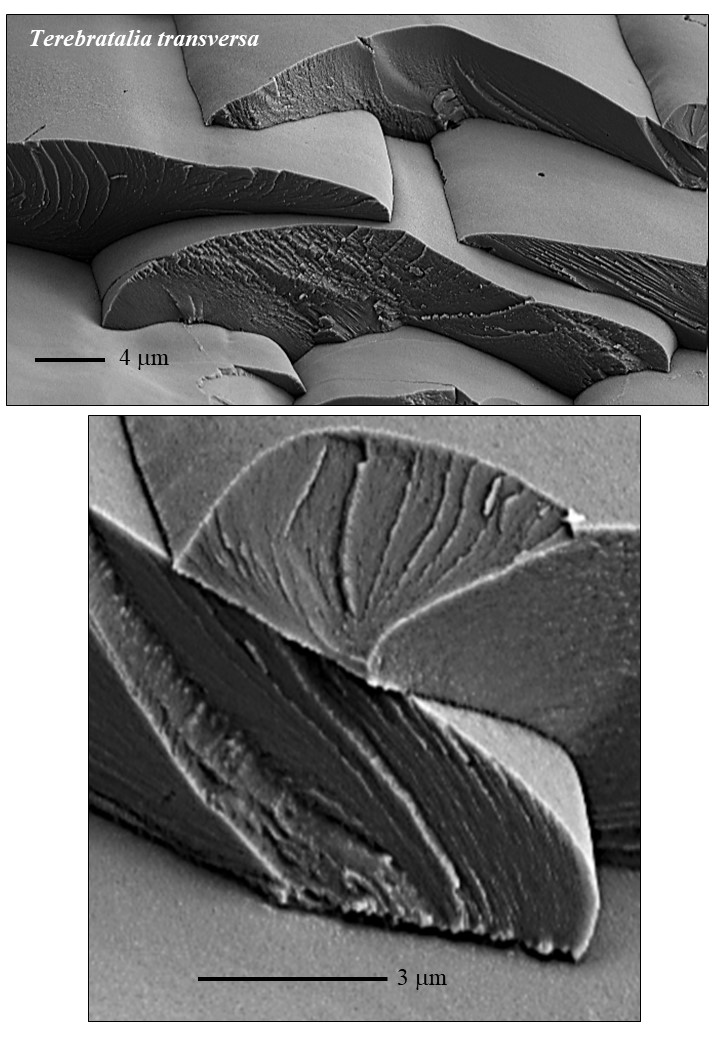


Figure S1. Conchoidal fracture of brachiopod fibrous calcite (*Terebratalia transversa*). See the characteristic curved conchoidal surface of fracture which is distinctly different to the smooth surface, obtained at brittle fracture. Compare the fracture surface of the sclerites of *S. ventrolineatus* and *F. gutturosus* (Fig. 5) with the fracture surface of *T. transversa* fibers.
